# Genetically Engineered Viral Vectors and Organic‐Based Non‐Viral Nanocarriers for Drug Delivery Applications

**DOI:** 10.1002/adhm.202201583

**Published:** 2022-08-15

**Authors:** Sakineh Hajebi, Satar Yousefiasl, Ilnaz Rahimmanesh, Alireza Dahim, Sepideh Ahmadi, Firoz Babu Kadumudi, Nikta Rahgozar, Sanaz Amani, Arun Kumar, Ehsan Kamrani, Mohammad Rabiee, Assunta Borzacchiello, Xiangdong Wang, Navid Rabiee, Alireza Dolatshahi‐Pirouz, Pooyan Makvandi

**Affiliations:** ^1^ Department of Polymer Engineering Sahand University of Technology Tabriz 51335‐1996 Iran; ^2^ Institute of Polymeric Materials Sahand University of Technology Tabriz 51335‐1996 Iran; ^3^ School of Dentistry Hamadan University of Medical Sciences Hamadan 6517838736 Iran; ^4^ Applied Physiology Research Center Isfahan Cardiovascular Research Institute Isfahan University of Medical Sciences Isfahan 8174673461 Iran; ^5^ Department of Anesthesia Jundishapur University of Medical Sciences Ahvaz 61357‐15794 Iran; ^6^ Department of Biology Faculty of Sciences University of Zabol Sistan and Baluchestan Zabol 98613‐35856 Iran; ^7^ Department of Health Technology Technical University of Denmark Kongens Lyngby 2800 Denmark; ^8^ Department of Chemistry Amirkabir University of Technology Tehran 15875‐4413 Iran; ^9^ Department of Chemical Engineering Sahand University of Technology Tabriz 51335‐1996 Iran; ^10^ Chitkara College of Pharmacy Chitkara University Himachal Pradesh 174 103 India; ^11^ Harvard‐MIT Health Science and Technology Cambridge MA 02139 USA; ^12^ Wellman Center for Photomedicine Harvard Medical School Boston MA 02139 USA; ^13^ Biomaterials Group Department of Biomedical Engineering Amirkabir University of Technology Tehran 15875‐4413 Iran; ^14^ Institute for Polymers, Composites and Biomaterials National Research Council IPCB‐CNR Naples 80125 Italy; ^15^ Department of Pulmonary and Critical Care Medicine Zhongshan Hospital Fudan University Shanghai Medical College Shanghai 200032 China; ^16^ School of Engineering Macquarie University Sydney NSW 2109 Australia; ^17^ Department of Materials Science and Engineering Pohang University of Science and Technology (POSTECH) 77 Cheongam‐ro, Nam‐gu Pohang Gyeongbuk 37673 South Korea; ^18^ Centre for Materials Interfaces Istituto Italiano di Tecnologia Pontedera Pisa 56025 Italy; ^19^ The Quzhou Affiliated Hospital of Wenzhou Medical University Quzhou People’s Hospital Quzhou Zhejiang 324000 China; ^20^ School of Chemistry Damghan University Damghan 36716‐41167 Iran

**Keywords:** abiotic nanomaterials, genetically manipulation, nanoparticles, non‐viral vectors, surface modifications

## Abstract

Conventional drug delivery systems are challenged by concerns related to systemic toxicity, repetitive doses, drug concentrations fluctuation, and adverse effects. Various drug delivery systems are developed to overcome these limitations. Nanomaterials are employed in a variety of biomedical applications such as therapeutics delivery, cancer therapy, and tissue engineering. Physiochemical nanoparticle assembly techniques involve the application of solvents and potentially harmful chemicals, commonly at high temperatures. Genetically engineered organisms have the potential to be used as promising candidates for greener, efficient, and more adaptable platforms for the synthesis and assembly of nanomaterials. Genetically engineered carriers are precisely designed and constructed in shape and size, enabling precise control over drug attachment sites. The high accuracy of these novel advanced materials, biocompatibility, and stimuli‐responsiveness, elucidate their emerging application in controlled drug delivery. The current article represents the research progress in developing various genetically engineered carriers. Organic‐based nanoparticles including cellulose, collagen, silk‐like polymers, elastin‐like protein, silk‐elastin‐like protein, and inorganic‐based nanoparticles are discussed in detail. Afterward, viral‐based carriers are classified, and their potential for targeted therapeutics delivery is highlighted. Finally, the challenges and prospects of these delivery systems are concluded.

## Graphical abstract

The organic and viral‐based genetically engineered carriers are classified, and their potential for targeted therapeutics delivery is highlighted. Also, the challenges and prospects of these delivery systems are concluded.

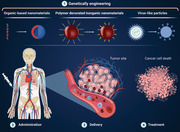

## 1 Introduction

Low solubility, rapid clearance, and adverse effects are the main limitations of conventional drug administration approaches. To overcome these inadequacies, various organisms and nanocarriers have been developed.^[^ ^1^, ^2^ ^]^ Nanotechnology has introduced operative nanoplatforms in drug delivery systems that have led to various progressive materials for the safe delivery and release of a wide variety of therapeutic agents into targeted sites.^[^ ^3^ ^]^ As a result, an ordered structure must be developed that could allow for a continuous release of the drug while overcoming first‐pass metabolism and pharmacological adverse effects. In recent years, nanostructures have been used to achieve continuous drug release with fewer adverse effects and a potent pharmacological response. Overexpression of the efflux pump, self‐repairing capability, modified targets, or enhanced drug metabolism all enable cancer cells developing a defense mechanism. These factors may have an impact on the treatment process, increasing the utility of specific nanocarriers that can bypass all of the defense mechanisms. These nanostructures are also used as a carrier molecule or transporter for vaccinations, drugs, deoxyribonucleic acid (DNA), proteins, and other substances.^[^ ^4^ ^]^ Although nanocarriers might carry various agents to the targeted sites, the design and synthesis of these nanomaterials show some disadvantages in pharmaceutical applications. As a result, a solution is required to improve the control of the particles.^[^ ^5^, ^6^, ^7^, ^8^ ^]^ For instance, the shape and size of modified carriers can be precisely controlled, which can offer the exact control on the attachment of drugs.^[^ ^9^ ^]^ Also, engineered materials can be used as noble candidates to greener, eco‐friendly, and high‐throughput systems to synthesize and assemble nanomaterials, even on the manufacturing scale.^[^ ^10^ ^]^ Additionally, genetic engineering propose control over the macromolecules used to construct nanocarriers. This ability permits exclusive properties such as specific biodegradation profiles and fully modified polymer and nanocarrier structures to be engineered and adjusted as required in some specific applications.^[^ ^11^ ^]^

Genetic engineering and bio‐fabrication are used as a unique method to synthesize organic nanoparticles such as protein‐based polymers, which can create structures with a thin molecular distribution. Different protein‐based polymers such as elastin‐based polypeptides can be synthesized using chemical methods. Though, some disadvantages get up when more complex structures require to be made, such as challenges related to purification and polydispersity and the effect of by‐products on the physical properties of the final product. The beginning of recombinant DNA technology and cloning techniques opened up a novel way in order to the manufacture of synthetic organic nanomaterials. This new assay intended several problems derived from the chemical synthesis methods, such as polydispersity, the need for organic solvents, and elimination of their residues.^[^ ^12^ ^]^ The development of drug delivery systems using such biopolymers, including silk‐elastin‐like proteins (SELP), silk‐like proteins (SLP), elastin‐like protein (ELP), and polysaccharides has improved significantly over the years.^[^ ^13^, ^14^ ^]^ Inorganic nanoparticles such as gold, silver, copper, iron oxide, and zinc, are biocompatible and nontoxic with considerably stable structure compared with organic nanomaterials. These nanomaterials are highly considered drug delivery carriers because of their great cellular uptake capability and low toxicity.^[^ ^15^, ^16^ ^]^ Functionalizing inorganic nanomaterials using genetically engineered polymer nanostructures can enhance their biocompatibility and bio‐functionality. The synthesis of plasmonic nanomaterials through genetic engineering of SELPs is an example of catalyzing a more comprehensive examination of the dynamic control in plasmonic nanoplatforms and provided a suitable carrier in drug delivery.^[^ ^17^ ^]^

Recently, the mixture of viral and bacterial nanocarriers with small molecules allowed encapsulating drugs and modified genetic materials into microorganisms. This assay permits the delivery of the therapeutic cargo encapsulated in the organisms to targeted sites due to the enchanting targeting potentials of these organisms.^[^ ^18^ ^]^ Virus‐like particles (VLPs), for example, have an effective transfer strategy for delivering their contents by mimicking the virus's mechanism. VLPs provide numerous benefits, including rapid synthesis, facile scaling, and high cell membrane penetration.^[^ ^19^, ^20^ ^]^ The probability of genetically modifying the proteins inside VLPs brands them a promising system to various applications. The viruses are effective immune activators and natural delivery vectors of genetic materials to their host; therefore, VLPs can be promising carriers for drug delivery.^[^ ^21^ ^]^ The different types of these engineered viral platforms are non‐enveloped VLPs, such as hepatitis B core antigen (HbcAg), cowpea chlorotic mottle virus (CCMV), cowpea mosaic virus (CPMV), tobacco mosaic virus (TMV), Johnson grass chlorotic stripe mosaic virus (JgCSMV), adeno‐associated viruses, P22, MS2, bacteriophage QB (*Qubevirus durum*), and reovirus that have been chemically or genetically engineered as drug delivery or vaccine carriers.

**Figure 1:**
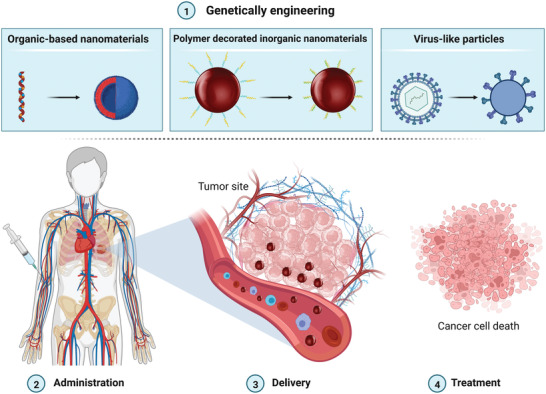
Schematic illustration of the synthesis of the genetically engineered organic, inorganic, and virus‐like nanostructures for drug delivery applications.

The current review focuses on drug delivery organisms and nanocarriers. An overview of the use of genetically engineered organic, modified‐inorganic, and viral nanostructures for drug delivery, as well as their frailties and advancements, are presented. In this review, we present a systematic overview of the recent developments and applications of genetically engineered nanoparticles in drugs, pro‐drugs, peptides, as well as nucleic acid components delivery. Ultimately, challenges and future tendencies in this area are emphasized, where such engineered assays will be of great importance and have the potential to be translated from benchtop to bedside (**Figure** **1**).

## 2 Genetically Engineered Compounds: Overview

Drug delivery systems can aid in the regulation of drug loading, controlled release, and release site, resulting in improved therapeutic outcomes and decreased toxicity and increased hydrophilicity, circulation time, and drug protection from undesired degradation.^[^ ^22^, ^23^, ^24^, ^25^ ^]^ Polymers are attractive options for drug delivery because of their ability to solve many strong drugs, stability, and chemical compounds via polymer chain alteration, copolymerization, block polymer length, and cross‐link with both side groups and the polymer backbone.^[^ ^26^, ^27^ ^]^ Generally, polymers fall into one of two categories such as natural polymers (starch, chitosan, gelatin, collagen, and albumin) and synthetic polymers (including polyorthoesters, polyesters, and polyanhydrides). Although synthetic polymers have physicochemical properties and high diversity, they have been challenged industrially for low compatibility, bulk hydrolysis, and acid degradation yields for drug delivery systems. As a result, using convergent science, focused research has been considered to overcome the mentioned limitations using natural polymers, genetic engineering, and biosynthesis.^[^ ^28^, ^29^ ^]^ In addition, with the development and complexity of drugs, the control of their properties and their transfer in the drug delivery systems require more accuracy that synthetic polymers cannot achieve more.^[^ ^30^ ^]^

Genetic engineering is used as a recombinant method to synthesize proteins or sometimes polysaccharide‐based polymers. This may make these structures have a narrow molecular weight distribution besides a well‐defined monomer composition, stereochemistry, and sequence. Genetically engineered protein‐based polymer nanocarriers possess advantages for gene/drug delivery such as biocompatibility, degradability without acidic degradation products, and homogeneity. Since chemical polymerization methods lead to the distribution of heterogeneous molecular weights of polymers, genetic engineering methods are considered for researches due to the synthesis of materials with homogeneous molecular weight distributions.^[^ ^31^, ^32^ ^]^ Therefore, polymers consisting of polypeptide chains provided through genetic engineering have useful and unique properties that with natural decomposition of these products, minimal toxicity is indicated. Another advantage of producing recombinant protein polymers is that they are homogenous and repeatable from batch to batch for use in the pharmaceutical industry. Recombinant proteins are biodegradable, stimuli‐responsive, have an increased therapeutic index, are targeted, have the ability to modify, can combine different amino acid sequence modules to create different libraries in amino acid synthesis, and have more detailed control over structure–function relationships. Recombinant proteins are biodegradable, stimuli‐responsive, have an increased therapeutic index, are targeted, have the ability to modify, can combine with different amino acid sequences to produce different amino acid libraries, and have more detailed control over structure–function relations.^[^ ^13^, ^33^ ^]^ However, in order to synthesize these genetically engineered polymer‐based proteins, first, a macromolecule (protein) is designed and then oligonucleotide (gene) encoding a protein is synthesized chemically and litigated into a recombinant vector. Finally, purification step is done. One of the most convenient methods is immobilized metal affinity chromatography.

Furthermore, the low cost of larger‐scale production in biomedical systems has resulted in the use of large‐scale recombinant protein polymers in a variety of important therapeutic applications, including drug delivery. Consequently, there has been an increased interest in recent years using recombinant protein‐based biopolymers such as SLP, ELP, and SELP in genetic engineering for the development of drug delivery systems. Inverse temperature transition actions, elasticity and amphiphilic, stabilization through physical cross‐links, and greater mechanical strength are all advantages of ELP‐ and SLP‐based engineered protein for drug delivery. Their communal features include enzyme‐controlled degradation, compatible, customizable chemistry and biomaterial characteristics, consistency in polymers, adaptable drug loading, and comparatively low‐cost construction and scale up.^[^ ^13^, ^34^, ^35^, ^36^ ^]^

Recently, cell membrane coating technology showed great consideration in the field of nanomedicine. Particularly, cell membrane‐coated nanoparticles, especially engineered NPs have established to be efficient drug delivery systems due to their long circulation times.^[^ ^37^, ^38^ ^]^ A novel assay in order to construct targeting ligands arises from the progresses in genetic engineering. This genetic engineering indicates the control of protein expression levels on the cell membrane through gene transfection. C1498 gene transfection for overexpression of VLA‐4 and extraction of the genetically modified membrane‐coated polymer nanoparticles core loaded with dexamethasone can be used to treat pulmonary inflammation. In fact, the biomimetic cell membrane exhibits a higher affinity for target cells that overexpress vascular cell adhesion protein 1 (VCAM‐1), thus increasing pulmonary inflammation more effectively by increasing the accumulation of VLA‐NP in the lung.^[^ ^39^ ^]^ In the following section, genetically engineered organic‐based macromolecules (such as proteins and polysaccharides) are represented. Then, genetically engineered organic‐based macromolecules coated inorganic materials are introduced.

## 3 Genetically Engineered Organic‐Based Nanoparticles

Natural polymers have received much attention in many medical fields as wound dressings, adsorbents, drug delivery, and medical scaffolds due to properties such as metabolic compatibility, nontoxicity, and resemblance to body tissue. The most important natural polymers in this regard are polysaccharides, proteins and polyamides, and polyesters. Methods used to produce natural polymers include:^[^ ^40^, ^41^ ^]^

- Direct use of biodegradable material (cellulose)
- Direct consumption of degradable animal polymers (collagen)
- Genetic engineering for better natural polymers (ELPs, SLPs)
- Microbial fermentation and access to materials such as polyhydroxyalkanoates

**Figure 2:**
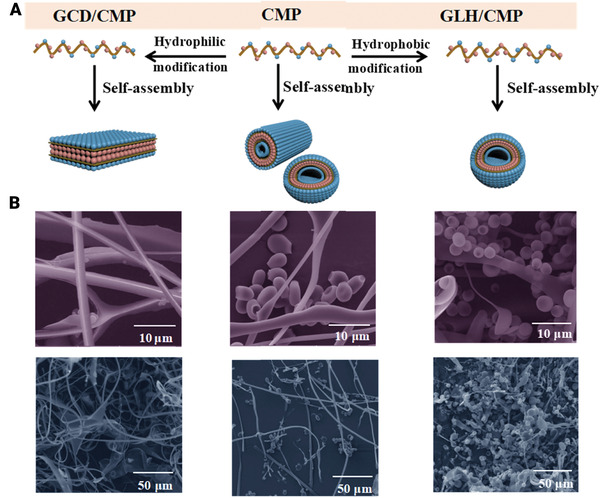
A) Schematic and B) representing SEM images of the self‐assembly of pure collagen (CMP), (GCD/CMP), (GLH/CMP). Reproduced with permission.^[^ ^43^ ^]^ Copyright 2021, Springer Nature. Abbreviation: CMP: Mimetic peptide; GCD/CMP: Glycidol‐modified CMP. GLH/CMP: Y‐glycidyl ether oxypropyl trimethoxysilane‐modified CMP.

Genetic engineering methods, which are the main focus of this review, will be discussed in detail in the following sections. Typically, nanosizing is a method to overcome poor aqueous solubility of drugs in pharmacology. Although cellulose and its derivatives have dimensions of 5–30 nm, they are widely used as nanocarriers in stabilization and drug delivery. For instance, Valo et al. have described a nanofibrillar cellulose matrix as stabilizer for nanoparticles to protect, store, and release hydrophobic drugs or genetically modified fusion proteins.^[^ ^42^ ^]^ At this work, anti‐solvent precipitation method was used to precipitate itraconazole, hydrophobic drug nanoparticle, in order to synthesis nanoparticles with either hydrophobics or genetically modified fusion protein and then binded to the cellulose. The results showed that by encapsulating the nanoparticles with cellulose, the processability and solubility rate were improved. Another type of recombinant polypeptide synthesized by genetic engineering technology is collagen mimetic peptide (CMP). CMP has numerous unique advantages including excellent structural controllability, high bioactivity, high biocompatibility, and elimination of animal‐derived viruses as well as wide range of functions. Luo and co‐worker investigated the self‐assembly mechanism of CMP using hydrophilic glycidol and hydrophobic Y‐glycidyl ether oxypropyl trimethoxysilane.^[^ ^43^ ^]^ The results revealed that the agent's hydrophilic interactions and hydrophobicity on CMP sub‐chains produce long‐term nucleation‐growth, which altered the structure and morphology of CMP (**Figure** **2**). The interaction through hydrophilic agents promotes the formation of nanofiber structures, whereas hydrophobic agents promote to microspheric structures, which could be beneficial in various biomedical applications.

**Figure 3:**
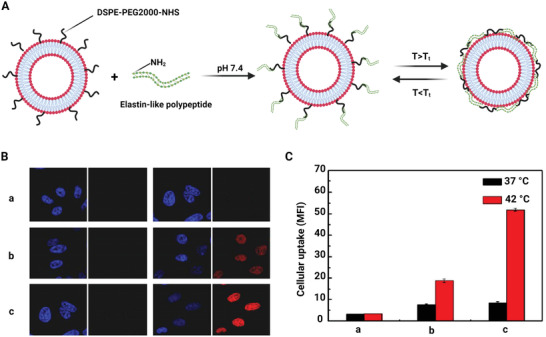
A) Synthesis of ELP‐modified liposomes. B) Confocal images of HeLa cells incubated in the culture media with DOX: a) Control, b) PEG‐liposomes, and c) ELP‐modified liposomes. C) Mean fluorescence intensity of liposomes identified through the flow cytometry assay: a) Control, b) PEG‐liposomes, and c) ELP‐modified liposomes. Mean and S.D. are shown (*n* = 3). Reproduced with permission.^[^ ^49^ ^]^ Copyright 2012, Elsevier. Abbreviation: ELP: Elastin‐like protein; DOX: Doxorubicin; PEG: Polyethylene glycol.

Elastin is one of the structural proteins present in connective tissue and has elastic properties. The amino acid in the structure of elastin is “hydroxyproline,” which is also present in the structure of collagen and forms a hydrogen bond and strengthens the structure of collagen.^[^ ^12^ ^]^ From this perspective, one of the synthetic biopolymers that have many applications in medicine and biotechnology is elastin‐like polypeptide. ELP is a synthetic biopolymer engineered and derived from the amino acid sequence contained in the hydrophobic parts of tropolastin, which has the ability to self‐aggregate as an intelligent polymer. The amino acid sequence of the elastin‐like polypeptide often consists of a repetitive sequence (palindrome) consisting of five amino acids VPGXG. X can be any amino acid except proline. The entry of proline alters the hydrophobic bonds to bind the ELP. ELP, on the other hand, is a heat‐reactive polypeptide that has a temperature‐dependent self‐aggregation property. For this synthetic protein, a reaction temperature is defined as the reverse transfer temperature (Reversible or Inverse Transition Temperature) or the minimum critical temperature of the solution.^[^ ^44^ ^]^ Fabrication formats (e.g., films and hydrogels) also improve utility of polymer‐based systems.^[^ ^45^ ^]^ ELP are thermally responsive polypeptides that are soluble in solutions at 37 °C, but which aggregate above 42 °C. Moreover, as drugs become more complex, the control of drug delivery becomes more difficult.^[^ ^46^ ^]^ Therefore, the properties required to match drug delivery demands need a level of customization that has not been achievable using synthetic polymer drug delivery systems. Unique features make ELPs perfectly suited to address current drug delivery challenges and lead to the development of ELP‐based therapies for the treatment of diseases—such as cancer and diabetes—and wound healing, and the development of bioengineering methods facilitates the design and fabrication of biocompatible, responsive, and multi‐faceted drug delivery systems. Many studies have been done on the thermosensitive ELP with reversible nano‐ and micro‐particles via coacervation.^[^ ^47^ ^]^ The processes of ELP phase transition, biodegradation, pharmacokinetics, and tumor localization in the nude mice with labeled method have been well‐described in a study by Liu et al.^[^ ^48^ ^]^ These researches were successfully labeled with two different ELPs with ^14^C; thermal responsive ELP1 showed that accumulation was higher in heated tumors than in cold tumors. ELP optimal molecular size determination is important because it affects the success rate of therapeutic agents to cross the tumor vessels. On the other hand, Na et al. have investigated enhanced cellular uptake of drug in tumor cells using synthase the ELP‐liposome‐drug conjugate (**Figure** **3**).^[^ ^49^ ^]^ They studied thermosensitive drug release properties and evaluated its cellular uptake efficiency under hyperthermic and normothermic conditions. Also, drug accumulation was carried out with flow cytometry and confocal microscopy inside tumor cells. The result has shown that higher ELP‐liposome‐drug conjugate internalization occurs in the ELP‐modified liposomes when compared to ELP‐unmodified liposomes. In vivo laboratory imaging results showed that intrarenal concentrations and distributions for ELP‐63 and ELP‐95 proteins are higher in the renal cortex due to their lower molecular weight, and the dose is approximately twice that of high‐molecular‐weight ELP proteins within the cortex. Other researches have shown that ELP‐conjugated cargo and their accumulation increase with repeated cycles of hyperthermia. Also, in hypothermic tissues, they have a lower toxicity than hyperthermic tissues. Therefore, with this method, they achieved controlled drug delivery due to the difference in their accumulation and removal in tissues in drug delivery.^[^ ^50^ ^]^

Another type of genetically engineered is silk‐like polymers (SLPs) with structures consisting of amoebic silk‐like blocks Gly–Ala–Gly–Ala–Gly–Ser. Actually, silk is a filamentous protein made by spiders and insects such as silkworms. There is a variety of silks that exist naturally. They have excellent mechanical properties, high strength, ductility, biocompatibility, and biodegradability. Also, silk materials are not limited to natural fibers, but some silk materials have been modified. Like chemically modified silk materials, composite materials and synthetic materials are decomposed and synthesized by enzymatic polymerization which can be a natural matrix effective for drug delivery.^[^ ^51^, ^52^ ^]^ Concerning genetically engineered silk‐like polymers, they have been mainly restricted to those designed on the repetition of the sequences [GGAGQGGYGGLGSQ‐GAGRGGLGGQGGAG] and [GPGGYGGPGQQGPGGYAPGQQPSGPGS] from the silk produced by the *Nephila clavipes* major ampullate glands 1 and 2, respectively.^[^ ^14^ ^]^ Some modifications of those base sequences have also been discovered. Some of them were applied to control the grade of crystallinity, though some other modifications have been added to functionalize the polymers, such as the incorporation of RGD cell attachment sequences. They commonly are deliberated to be block copolymers with greatly conserved repeats of short side‐chain amino acids as hydrophobic blocks and short sequences.^[^ ^14^ ^]^

Because polymers consist of only silk blocks with very low aqueous solubility and low flexibility, copolymerizing them with elastin‐like blocks increases their flexibility and hydrophilicity while reducing total crystallization. Block copolymers such as SELPs are obtained using genetic engineering techniques that can be used to control physicochemical properties by modifying the composition and sequence of the copolymer.^[^ ^36^ ^]^ In general, the structure of these polymers is denoted by the symbol, where they are a silk‐like block denoted by s, which is composed primarily of GAGAGS repeats, and elastin‐like sequences with the VPGVG amino acid sequence, denoted by the symbol E.^[^ ^53^, ^54^ ^]^ The potential uses of these polymers in drug delivery systems and tissue engineering are under study. Although, in spite of the growth in studies with SELP, little is recognized about the crucial self‐assembly properties of these polymers.^[^ ^55^ ^]^ However, increasing the number of repeats of elastin blocks reduces the crystal structures of silk and leads to an increase in water solubility of the entire SELP block at low temperatures. This property is very important for purification of protein polymers and pharmaceutical formulations. Therefore, in drug delivery applications, this SELP with the interactions of hydrophobic blocks and the formation of micelle core in water with self‐assembly force as nanocarriers have been highly considered by researchers.^[^ ^56^, ^57^ ^]^

**Figure 4:**
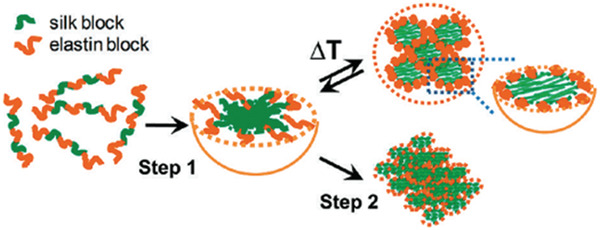
Self‐assembly of micellar‐like nanoparticles in T‐responsive. Reproduced with permission.^[^ ^58^ ^]^ Copyright 2011, American Chemical Society.

**Figure 5:**
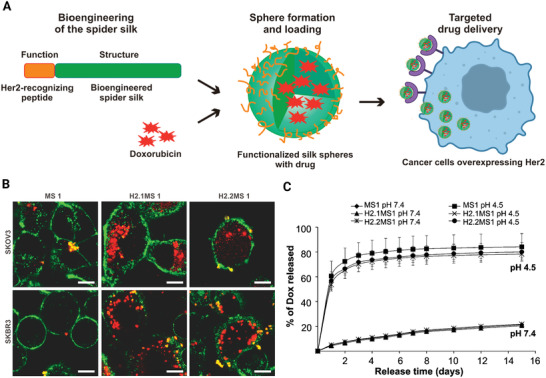
A) Bioengineering of the spider silk. B) Cellular uptake of the silk spheres on the SKOV3 and SKBR3 cells, which were treated with spheres prepared of fluorescently labeled control MS1 and silk proteins H2.1MS1 and H2.2MS1. Scale bar = 10 µm. C) Behavior of release of DOX. Reproduced with permission.^[^ ^59^ ^]^ Copyright 2014, American Chemical Society.

By regulating the ratio of elastin to silk blocks, various shapes such as nanoparticles, hydrogels and nanofibers can be produced reversibly or irreversibly. Due to the outstanding physical and unique properties of silk and elastane copolymers, they have been extensively studied in the fields of tissue engineering and drug delivery. Xia et al. have investigated the self‐assembly behavior of SELPs by adjusting the ratio of silk‐to‐elastin blocks in two stages.^[^ ^58^ ^]^ The results showed that increasing temperature and the resulting hydrogen bonding and hydrophobic interactions led to nanostructures with reversible or irreversible properties self‐assembled. They for the first time showed that recombinant SELPs under a two‐step self‐assembly process in aqueous solutions were first formed by hydrogen bonding between blocks to form micellar‐like particles with mainly silk block GAGAGS which are hydrophobic units as the core structure and elastin blocks as corona, which can be controlled by adjusting the ratio of silk blocks to elastin (**Figure** **4**). In the second stage, the behavior of these micelles in response to temperature was investigated. By fine‐tuning the ratio of silk blocks to elastin, various structures such as nanoparticles, hydrogels, or nanofibers were produced in a reversible or irreversible manner. Since it was found that Her2 overexpression in tumors is higher than that of healthy tissue, a new approach is developed for agents targeting the Her2 in cancer treatment. In this ELP field, Florczak et al.^[^ ^59^ ^]^ designed various hybrids of spider silk with binding domain different from Her2 receptor by genetic engineering to control the drug delivery systems (**Figure** **5**). The behavior of release of doxorubicin (DOX) on the aqueous solution properties of MS1, H2.1MS1, and H2.2MS1 spheres was investigated. The results showed that release rate at pH 4.5 were faster than it was at pH 7.4.

One of the most significant parameters that can be used to change the properties of copolymers is how the sequence of monomers is located in the copolymers. The polymerization procedure and the sequence distribution of various monomer units in the copolymer depend on the placement, reaction ratio, and instantaneous composition of the monomer. The sequence distribution of monomers or amino acids by controlling the polymerization temperature, concentration of reactants, and the process can lead to a variety of copolymers.^[^ ^60^, ^61^ ^]^ Recombinant DNA methods are one of the techniques to attain precise control over macromolecular structure. Protein polymers synthesized using genetic engineering contain amino acids such as sequential polypeptides and poly (amino acids), which are homopolymers or random copolymers made by polymerizing an amino acid or a mixture of amino acids.^[^ ^62^ ^]^

Micelles with hydrophilic blocks (poly L‐amino acid) and hydrophobic (poly ethylene oxide) composed of block copolymers using chemical methods or biosynthesis have been proposed as drug carriers to increase blood circulation.^[^ ^63^ ^]^ However, due to the lack of resistance of these polyethylene's, the application caused a number of limitations. The development of polyamide‐derived biomass as an engineered biopolymer has created an emerging interest in sustainable chemistry and engineering. High‐performance natural materials, including silk and elastin, are composed mainly of poly (amino acids) and small amounts of short peptides, suggesting that poly (amino acids) could be substituted for oil‐derived polymers. However, for the creation and development of biomass‐based polymers such as silk and elastin, the synthesis and design of poly (amino acid) materials is under consideration.^[^ ^64^ ^]^ This synthetic methodology allows the precision of genetic protein synthesis in the synthesis of new macromolecular structures for drug delivery. The following are examples of the application of these polymers in the field of drug delivery, such as ELPs, ELPBCs, SELPs. **Table** **1** reports sample ELPs/SELP‐based sequences for drug delivery applications.^[^ ^65^, ^66^, ^67^, ^68^, ^69^, ^70^, ^71^, ^72^, ^73^, ^74^ ^]^

**Table 1:** Samples Elastin‐like protein (ELPs)/silk elastin‐like protein (SELP)‐based sequences in the drug delivery fields

Functions	Material Format	Drug/targeting	Ref.
ELPs	Micelles	Anticancer cell	[65]
SELP‐47K	Film	Antibiotic/Ciprofloxacin	[66]
SELP	Matrix‐metalloproteinase	Doxorubicin	[67]
ELPs	Hydrogel	Wound repair	[68]
ELPBCs	Micelle	Anticancer drug	[69]
ELP	Gel‐like	Radionuclide	[70]
Elastin‐mimetic polypeptide	Tailor‐made	Doxorubicin and rhodamine‐GFLG	[71]
ac‐ELP‐H1	Particle	c‐My	[72]
ELP	Particle	Doxorubicin	[73]
SynB1–ELP	Particle	Doxorubicin	[74]

SELP, silk‐elastin‐like proteins; SLP, silk‐like proteins; ELP, elastin‐like protein; ELPBCs, elastin‐like polypeptide diblock copolymers.

**Figure 6:**
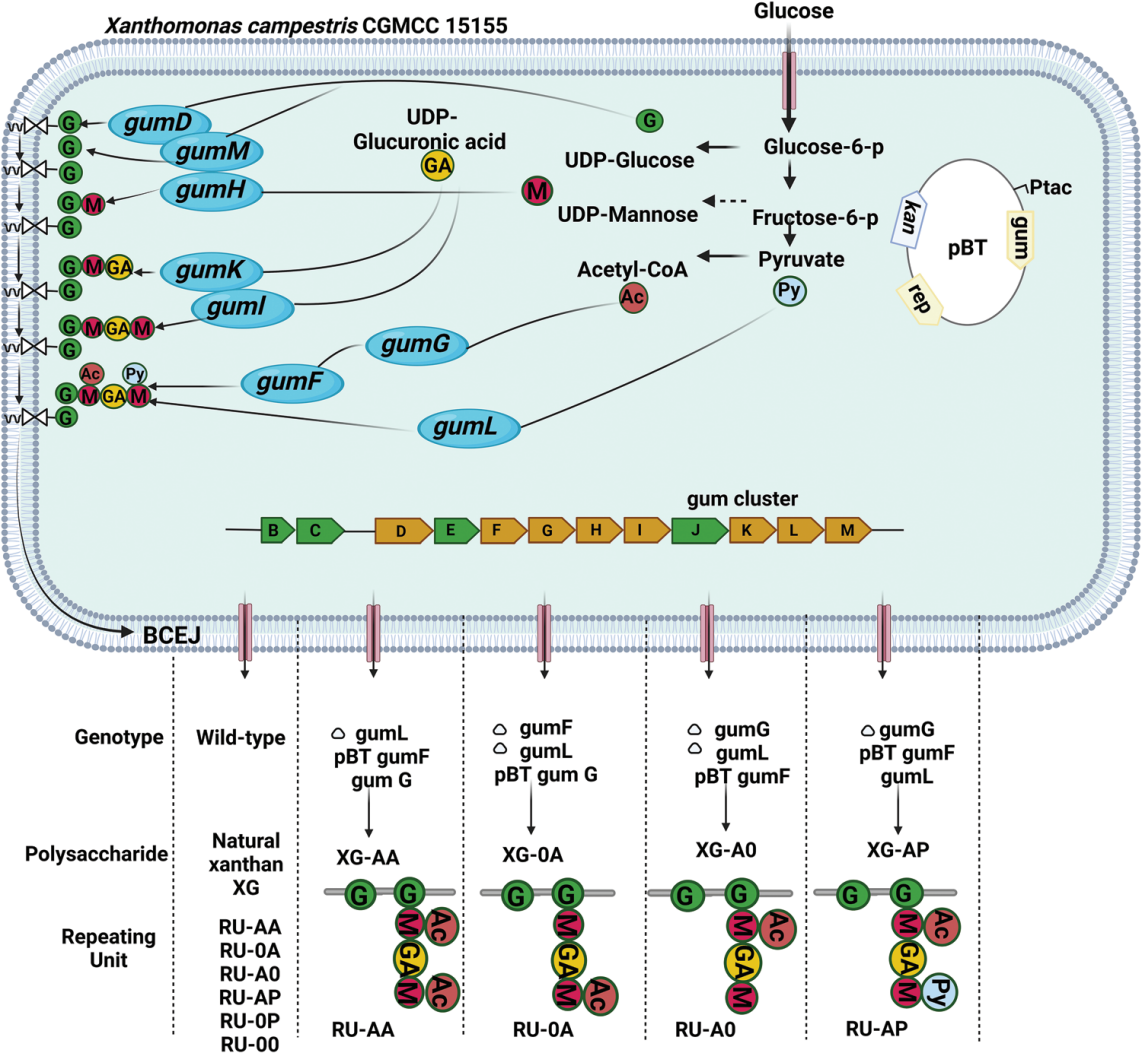
The primary structures of customized xanthan in *Xanthomonas campestris* and their biosynthetic pathways.^[^ ^80^ ^]^

Tailor‐made polysaccharide depending on multiple structures has been offered as a novel concept in recent years, which will open new windows for biomaterial universe.^[^ ^75^, ^76^ ^]^ Xanthan gum is an essential food ingredient and is produced by *Xanthomonas campestris* (*X. campestris*).^[^ ^77^, ^78^ ^]^ It has a cellulose‐like backbone with a (3 1) linked ‐d‐Man‐(2 1) chain, ‐*β*‐d‐GlcA‐(4 → 1). Every second glucose unit has a —d‐Man side chain (Man, mannose; GlcA, glucuronic acid).^[^ ^79^ ^]^ In a recent study, eight polysaccharides have been synthesized with uniformly distributed repeating units and different rheological properties (**Figure** **6**).^[^ ^80^ ^]^ The synthesized polysaccharides were produced in *X. campestris* CGMCC 15155 using marker less gene knockout and gene overexpression methods. The findings revealed that their distinct homogenous primary structures controlled their individual secondary structures and rheological features, particularly the terminal mannose, pyruvyl group, and acetyl group connected to the internal mannose of the side chain. Both the terminal mannose residue and the inner acetyl group might stabilize the double helix structure; however, the terminal mannose residue improved while the internal acetyl group decreased solution viscosity and modulus.

## 4 Genetically Engineered Organic Compounds Decorated Inorganic Nanoparticles

Inorganic nanomaterials have been extensively investigated in biomedical sciences for the past few decades due to their exciting physicochemical properties. Through synthesis of various kinds of nanomaterials and modifying them, new horizons can open up for exciting biomedical applications. For instance, modified nanomaterials are employed for magnetic separation, as carrier for targeted drug and gene delivery, and diagnostic imagining. For instance, by giving them a magnetic resonance contrasts or making them fluorescent it is easy to track them once implanted in the body.^[^ ^5^, ^24^ ^]^ Generally, this kind of nanomaterials contain two parts: metallic nanoparticles constitute the core part, and polymeric (mainly) biomaterials are used in the shell or surface part. On the one hand, these methods arrange a proper physical layer for conjugation of bio macromolecules; on the other hand, they prevent unwanted physiochemical interaction in the body.^[^ ^81^, ^82^ ^]^ Furthermore, their stability and protection from microbial attack, by comparison with organic nanoparticles, has made them even more important for biomedical applications. Inorganic nanomaterial can be classified in four separate groups based on their origin: metal‐based nanoparticles, carbon‐based nanoparticles, ceramic nanoparticles, quantum dots metal‐based nanoparticles. Metal‐based nanoparticles, such as gold, silver, copper, iron oxide, and zinc, have attracted scientist over a century. Synthesizing nanoparticles in different shapes (nano cage, nano shell), size, and their surface functionalization, which provides a wide range of potential applications, can be named the most important advantages of this class of biomaterials.^[^ ^83^, ^84^, ^85^ ^]^

**Figure 7:**
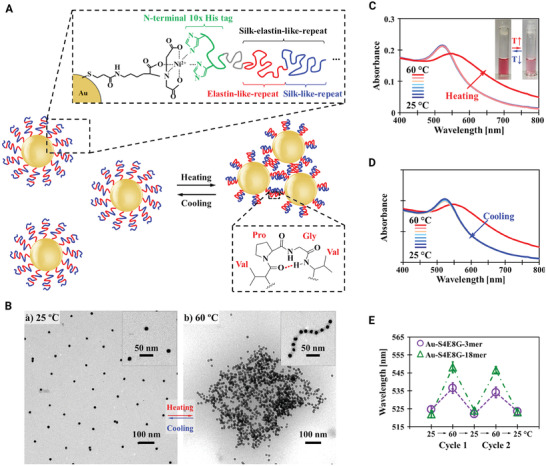
Thermoresponsive plasmonic gold/silk‐elastin protein core–shell nanoparticles. A) Schematic of functionalized gold nanoparticles with SELPs. B) (a) Representative TEM images of functionalized gold nanoparticles in the individual form at 25 °C and (b) in aggregated form when heated to 60 °C. C,D) Variable‐temperature UV−vis extinction spectra of the functionalized gold nanoparticles at room temperature and when heated to 60 °C. E) Thermal cycles of functionalized gold nanoparticles between 25 and 60 °C were monitored by UV−vis spectroscopy indicating good reversibility. Reproduced with permission.^[^ ^17^ ^]^ Copyright 2014, American Chemical Society.

For many years metallic nanoparticles have been investigated for various biological applications including, bioactive delivery, drug delivery, and preconcentration of target analytes, as a vehicle for gene delivery, and imaging.^[^ ^86^ ^]^ Generally, metallic nanoparticles can be categorized into noble metal nanoparticles (especially gold and silver) and magnetic nanoparticles (MNPs) (iron oxide). Both have promising features that draw attention to themselves and reduce side effects of conventional treatments. Noble metallic present a great application in optical, imaging fields, photo thermal therapy on account of their surface plasmon resonance. For instance, gold nanoparticles (AuNPs) are used in therapeutic strategy due to their ability to convert light into heat. An example includes AuNPs that have been injected into tumor systematically or locally to increase the temperature to a sufficient level under laser irradiation, as a consequence inducing localized cell death. In addition, these nanoparticles possess an excellent antimicrobial activity toward numerous microorganisms, such as bacteria, fungi, and viruses. Even though this raises concerns regarding the affection on healthy human cells by various mechanisms, burgeoning technology has allowed synthesizing them in a novel method to reduce their adverse effects on human bodies.^[^ ^87^, ^88^ ^]^ Numerous studies have been reported on the use of genetically engineered polymers modified with inorganic nanomaterials. To tune the temperature response in plasmonic gold, genetic engineering has been used to design thermo‐responsive and photonic core–shell nanoparticles. Using gold nanoparticles as core, researchers synthesized SELP polymers with amino acid sequences [(GVGVP)4(GGGVP)(GVGVP)3(GAGAGS)4] as shells and investigated temperature response on self‐assembly of the SELP shell. It was indicated that the *β*‐silk structure stabilizes the structure of nanoparticles in response to the temperature. Also, with increasing temperature to 60 °C, nanoparticles accumulate and form a square state (**Figure** **7**).^[^ ^17^ ^]^

MNPs display a unique magnetic and heat‐medicated inherent as well as being biodegradable and biocompatibility. Due to their innate characteristics, MNPs are easily used in targeted drug delivery and hyperthermia‐based therapies. Hyperthermia based on the heat that these nanoparticles generate in an alternating magnetic field.^[^ ^89^, ^90^, ^91^ ^]^ Furthermore, by applying external magnetic fields and under specific conditions, the drug loaded on MNP can be directed to specific point in body, and accuracy of it depends on the depth of the targeted part. Magnetite (*γ*‐Fe_2_O_3_) and magnetite (Fe_3_O_4_) superparamagnetic nanoparticles are the major MNPs used in drug delivery.^[^ ^91^ ^]^

A magnetic responsive system was developed using ELPs and superparamagnetic iron oxide nanoparticles (SPIONs). The magnetic characteristics were attained by entrapping SPIONs in the polymeric matrix to manage microparticle targeting and monitoring. The results showed that prepared microparticles showed a round shape and brown color, suggesting the successful encapsulation of SPIONs in the polymeric matrix. Furthermore, fabricated microparticles demonstrated cytocompatibility toward in vitro cell model and indicated efficient drug encapsulation with a two‐phase release profile. Taken together, this study highlighted the drug delivery potential of these systems for various biomedical applications.^[^ ^92^ ^]^

Recently, carbon allotropes: fullerenes, carbon nanotubes (CNTs), graphene oxide (GO), carbon and graphene, quantum dots, and their size, shape, and surface properties, as well as antimicrobial behavior, have received tremendous attention and have opened up a gateway for pharmaceutical applications.^[^ ^93^ ^]^ Furthermore, a significant part of the human body comprises carbon, so it can be said to be a biocompatible material. The application as a drug delivery system is common in carbon‐based nanoparticles; they can be used to carry biological molecules including DNA, proteins, and drugs. Drug compounds are loaded on the surface or inside these structures. Targeting and simultaneous transfer of two or more compounds are other features of these particles in drug delivery; especially the graphene‐based nanoparticles have a remarkable position in this area due to their unique structure, high surface area, and existence of several functional groups, including carboxyl, epoxy (in GO), suggesting an excellent loading capability for a variety of fluorescent probes and drugs.^[^ ^94^ ^]^

**Figure 8:**
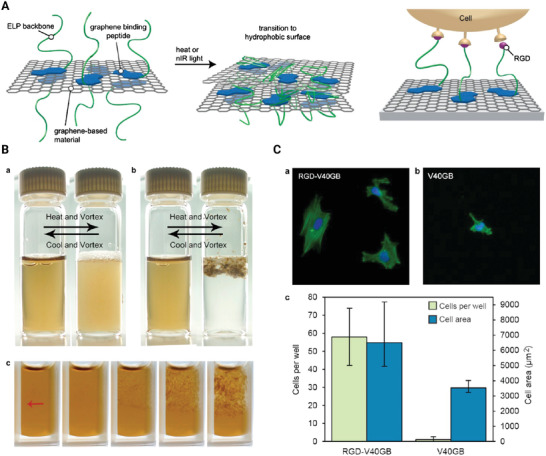
A) Schematic illustration on the preparation of stimuli‐responsive graphene‐based materials functionalized with elastin‐like polypeptides. B) Images of ELP(V50)‐treated graphene oxide solutions before and after the inverse temperature transition (a); image of significant and reversible aggregation of ELP(V50GB)‐treated graphene oxide after the inverse temperature transition (b). c) The nIR‐induced aggregation of ELP(V50GB)‐treated graphene oxide is shown in a series of photos taken over the course of 4 min. The arrow indicates the nIR beam's position. C) Fluorescent images of cells remaining on a) RGD‐ELP(V40GB)‐treated reduced graphene oxide and b) ELP(V40GB)‐treated reduced graphene oxide. c) Average cell number and area of cells on treated reduced graphene oxides. Reproduced with permission.^[^ ^95^ ^]^ Copyright 2014, American Chemical Society. Abbreviation: ELP: Elastin‐like protein.

Elastin‐like polypeptide was used to functionalize graphene‐based nanomaterials in order to investigate stimuli‐responsiveness behavior and control their physical, colloidal properties such as bioactivity of graphene. By combining the thermal responsiveness of ELP with photothermal capabilities of GO sheets, the fabricated hybrids demonstrated responsiveness toward nIR light. Furthermore, the ELP coating inhibited the aggregation properties of GO sheets at higher concentration and enabled the dispersion in organic solvents. Such graphene–protein nanocomposite materials showed promising stimuli‐responsive properties for drug delivery application (**Figure** **8**).^[^ ^95^ ^]^

## 5 Nanoscale Genetically Engineered Viral Platforms

**Figure 9:**
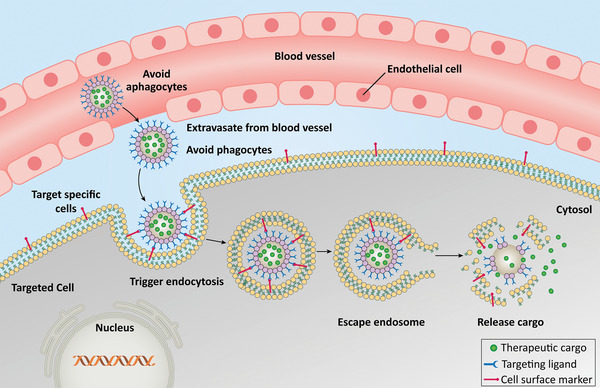
Virus‐like particles (VLPs) act as highly effective drug delivery vehicles. The surface of VLPs modified and functionalized with biomolecules. These ligands can be used to target specific cells, which can overcome the numerous challenges with delivering therapeutic cargo. After extravasation, VLPs target specific cells and trigger internalization while escaping the immune system. The VLPs escape the endosome after endocytosis and subsequently disassemble to release their cargo.

Nanotechnology facilitated the way, which we prevent, detect, and cure diseases. This progress has been made feasible by viruses, which can operate as prefabricated nano‐scaffolds with unique features that can be easily modified.^[^ ^96^ ^]^ Viruses are called natural nanomaterials as they have a genetically content, such as nucleic acids, encircled through a protein coat capsid and are in the size of 20–500 nm. The removal of genetic biomaterial results in an empty capsid that can be used to host drugs, enzymes, antibodies, or aptamers. These genetic engineering leads to precision drug delivery and targeted therapy. VLPs are nanoscale constructs composed of assembled viral proteins that are non‐infectious because they lack viral genetic material. The viral structural proteins can be expressed and self‐assembled in a variety of live or cell‐free expression methods, after which the viral structures can be assembled and reconstructed. VLPs have unique properties that allow them to create innovative platforms for medicine and biotechnology by combining techniques from each of these disciplines.^[^ ^97^ ^]^ The following sections describe some chemically or genetically engineered VLPs that have been used as a vehicle for drug delivery or vaccine carriers. Recently different ligands have been functionalized to VLPs, and their targeting capacity has been examined in vitro and in vivo, which have been proposed to use as vehicles for cargo delivery as shown in **Figure** **9**.

Viral capsids are most attractive natural structures for variety of biological applications due to their extreme stability in high temperatures, across a wide range of pH, and in organic solvent–water mixtures. Because of these characteristics, as well as their inherent biocompatibility, viruses have been developed as actively targeted delivery platforms, which can improve recent drug delivery methods.^[^ ^98^, ^99^ ^]^ In the current section, different nanostructures based on genetically engineered viruses and their application in drug delivery will be reviewed.

### 5.1 MS2

**Figure 10:**
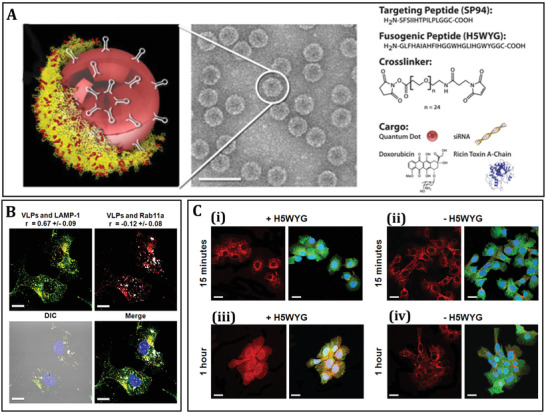
Bacteriophage MS2 virus‐Like particle‐based cell‐specific delivery induces selective cytotoxicity in tumor cell. A) The procedure of developing HCC‐specific MS2 VLPs that encapsulate different therapeutic and imaging components is depicted in this diagram. Using a suitable cross‐linker, nanoparticles (e.g., quantum dots), protein toxins, and DOX are attached to the pac site. Targeting peptides can be added to cargo‐loaded VLPs to increase selective internalization by cancer cells, fusogenic peptides can be added to promote endosomal escape of internalized VLPs, and PEG can be added to minimize nonspecific interactions and the humoral immune response to coat protein. Using a heterobifunctional cross‐linker with a PEG spacer arm, peptides having a C‐terminal cysteine residue are attached to lysine residues (red) on the outer capsid surface (yellow). B) Internalization of Alexa Fluor 555‐labeled SP94‐targeted VLPs (red) occurs via an endocytotic pathway, as evidenced by their punctate presence within HCC. The positive Pearson's correlation coefficient (*r*) between SP94‐targeted VLPs and Alexa Fluor 488‐labeled LAMP‐1 (green) as well as the near‐zero *R*‐value between SP94‐targeted VLPs and Alexa Fluor 647‐labeled Rab11a (white), a marker for recycling endosomes, show that SP94‐targeted VLPs are directed to lysosomes upon endocytosis by HCC. C) Hep3B cells were treated to SP94‐targeted VLPs (red) for 15 min or 1 h. In (i) and (iii), VLPs were co‐modified with SP94 peptide and H5WYG peptide, while in (ii) and (iv), VLPs were co‐modified with SP94 peptide alone. VLPs co‐modified with the SP94 targeting peptide and the H5WYG fusogenic peptide get disseminated in the cytoplasm of Hep3B cells after endocytosis, but VLPs changed with just SP94 remain in endosomes. Alexa Fluor 555 was used to label VLPs. Hoechst 33342 and CellTracker Green CMDFA were used to label the cells. Scale bars = 10 µm. Reproduced with permission.^[^ ^101^ ^]^ Copyright 2011, Elsevier. Abbreviation: HCC: Human hepatocellular carcinoma; LAMP‐1: Lysosomal‐associated membrane protein 1; Rab11a: Ras‐related protein Rab‐11A.

MS2 bacteriophage‐derived VLPs are icosahedral capsids with 22–29 nm in diameter and are formed by self‐assembling 180 copies of a single coat protein (CP). These VLPs are a unique delivery platform, and their desirable properties made them a popular research field in recent years due to many reasons. First, these can be used as a carrier for bacteriophage capsids to transfer epitope peptides, Ribonucleic acid (RNAs) or DNAs, and drugs in a cost‐effective and convenient manner. Second, they can act as excellent adjuvants, inducing both arms of immune responses.^[^ ^100^ ^]^ Because of their rapid assembling nature in the presence of nucleic acids, researchers succeeded to encapsulate DOX, cisplatin, 5‐fluorouracil, siRNA cocktails, and protein toxin (ricin toxin A‐chain (RTA)) inside modified MS2 VLPs. Similarly, MS2 VLPs have been used for the selective distribution of chemotherapeutic drugs human hepatocellular carcinoma cells (HCC) after functionalizing with a displayed targeting peptide (SP94). At sub‐nanomolar drug concentrations, all drug formulations tested with this platform resulted in selective cytotoxicity. This experiment used MS2's potential to encapsidate nucleic acids to demonstrate that delivering different anti‐tumor agents within VLPs caused cancer cells growth arrest and apoptosis. These modified VLPs had a great affinity for HCC cells, with limited uptake in healthy cells, and have a cytotoxic effect at minimal dosage. The SP94‐targeted VLPs encapsidated with drugs such as DOX, cisplatin, and 5‐fluorouracil have shown selective destruction of the HCC cell line Hep3B even at the lowest concentration (1 nm) of drug loading. In addition, at <150 pm concentration of siRNA, encapsidated SP94‐targeted VLPs suppressed the expression of cyclin family members, and induced growth arrest and apoptosis in Hep3B. Similarly, without damaging the control cells, nearly 100% of Hep3B cells are killed by MS2 VLPs loaded with RTA (at a concentration of 100 fm) and modified to co‐display SP94 targeting peptide and a histidine‐rich fusogenic peptide (H5WYG) that promotes endosomal escape. Because of their tolerance of multivalent peptide display and capacity to selectively encapsidate a range of cargos, MS2 VLPs induce selective tumor cytotoxicity in vitro (**Figure** **10**).^[^ ^101^ ^]^

### 5.2 MSN‐SH

**Figure 11:**
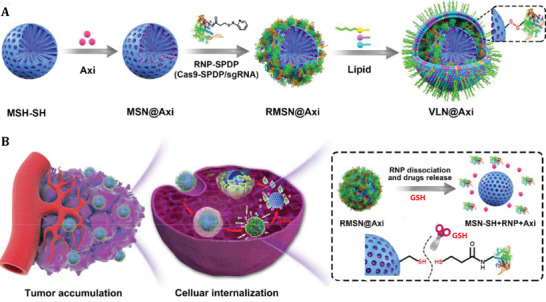
Schematic illustration of axitinib‐loaded VLN synthesis. A) Axitinib and the CRISPR/Cas9 system are loaded into the mesoporous silica nanoparticle (MSN)‐based core, which is further enclosed with a lipid shell in VLN's core–shell structure. B) In response to the reductive microenvironment, VLN releases the CRISPR/Cas9 system and drug. Reproduced with permission.^[^ ^102^ ^]^ Copyright 2020, Elsevier. Abbreviation: GSH: Glutathione; Axi: Axitinib; VLN: Virus‐like nanoparticle.

VLPs have recently been used as an effective vector for delivering the CRISPR/‐Cas9 system (clustered regularly interspaced short palindromic repeats [CRISPR]‐associated protein 9 [Cas9]) for cancer gene therapy. In this study, researchers used a virus‐like nanoparticle (VLN) as a flexible nanoplatform for co‐delivering the CRISPR/Cas9 system and biomolecule for effective tumor therapy. In order to construct VLN, axitinib, a small molecule of tyrosine kinase inhibitor, is loaded into the pores of surface‐thiolated mesoporous silica nanoparticles (MSN‐SH) and the pores were then sealed using disulfide bonds to link ribonucleoprotein (RNP) to MSN‐SH (RMSN). Afterward, VLN was produced by encapsulating RMSN with a lipid layer containing PEG2000‐DSPE, which prevents enzymatic degradation of RNP in the physiological condition and extends half‐life in the circulation. VLN releases RNPs and small molecules in response to the reduced microenvironment inside tumor cells, leading in the combinatorial modulation of several signaling paths (**Figure** **11**).^[^ ^102^ ^]^

A CRISPR/Cas9 with a single guide RNA (sgRNA) targeting the programmed death‐ligand 1 (PD‐L1) encoding gene (sgPD‐L1) was used to disrupt the immunosuppressive tumor microenvironment and increase the effectiveness of VLN in cancer therapy.

In vitro and in vivo experiments have shown that the tumor growth was effectively suppressed in cancer cells using VLN by disruption of pathway of the PD‐1/PD‐L1 and reinvigoration of exhausted T cells. Additionally, VLN was capable of delivering Axi to the tumor, leading in a reduction in the number of regulatory T cells (Tregs) in the tumor microenvironment. T‐cell‐mediated anti‐tumor immunity was increased when immunosuppressive Tregs were reduced, and as a result, tumor development was inhibited.

**Figure 12:**
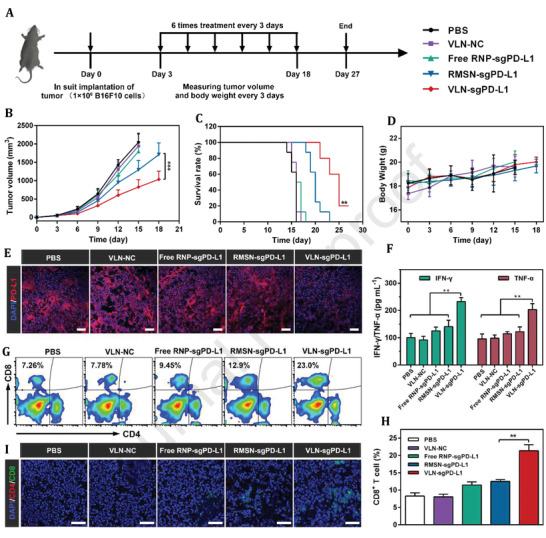
The VLN‐sgPD‐L1 anti‐tumor activity in melanoma mice model. A) Administration timelines and procedures for various formulations. B) Tumor growth curves in PBS, VLN‐sg (carrying a sgRNA without PD‐L1 targeting sequence), free RNP‐sgPD‐L1, RMSN‐sgPD‐L1, and VLN‐sgPD‐L1 mice. C) Survival curves for mice received several formulations. D) Body weight changes in mice after treatment with various formulations. E) Analysis of PD‐L1 immunofluorescence in each treatment group. Alex Fluor 594 (red) and DAPI (blue) were used to counterstain PD‐L1 and nuclei, respectively. Scale bars = 100 m. F) Levels of IFN‐ʏ and TNF‐*α* in mice treated with various formulations. Representative plots (G) and quantitative analysis (H) of CD8+ tumor infiltrating lymphocytes infiltration in treated tumors assessed by flow cytometry (gated on CD45+ CD3+ cells). I) Immunofluorescence image of the tumors revealed infiltrating CD4+ T cells (red) and infiltrated CD8+ T cells (green). The nuclei of the cells were counterstained with DAPI (blue). Scale bars = 100 µm. Data are presented as mean ± s.d. The significant levels are shown as **p < 0.01, and *** *p* < 0.001. Reproduced with permission.^[^ ^102^ ^]^ Copyright 2020, Elsevier. Abbreviation: VLN; Virus‐like nanoparticle; sgRNA: Single guide RNA; PBS: Phosphate buffered saline; PDL‐1: Programmed death‐ ligand‐1; INF: Interferons; TNF: Tumor necrosis factor; RNP: Ribonucleoprotein; MSN: Mrsoporous silica nanoparticle.

By delivering Cas9 protein, sgRNA, and small molecule, VLN exhibited synergistic control of multiple pathways in tumors, demonstrating VLN's potential as a generic platform for the development of advanced combination therapies against malignant tumors (**Figure** **12**). These results show that VLN is able to deliver gene/drug systems to tumor tissue more efficiently, and provide a solid basis for efficient gene editing in tumors and reduction of side effects in normal tissues.^[^ ^102^ ^]^

### 5.3 PhMV

The use of nanoparticle‐based delivery vehicles to control drug release might improve chemotherapy safety and effectiveness. Using a combination of chemical conjugation and stacking interactions the prodrug 6‐maleimidocaproyl‐hydrazone DOXDOX‐EMCH) was loaded into the empty core of VLPs derived from Physalis mottle virus (PhMV). After cellular absorption, an acid‐sensitive hydrazine linker present in the DOX‐EMCH prodrug releases the doxorubicin in the slightly acidic tumor microenvironment or acidic endosomal/lysosomal compartments. To reduce nonspecific absorption and promote biocompatibility, the exterior surface of VLP was coated with polyethylene glycol (PEG). The DOX‐EMCH molecule's pH‐sensitive hydrazone bond permits DOX to be released in the tumor microenvironment or after absorption into the endosome/lysosome. After incubation at 37 °C for 48 h, the higher DOX was released at pH 5.2 (67%) than at pH 6.4. While quicker and efficient DOX release from the DOX‐PhMV‐PEG particles was observed under conditions that mimicked the tumor microenvironment or endosome/lysosome, the cargo was retained during normal circulation. On the other hand, the release profile plateaued after 48 h at pH 7.4 with less than 10% of the drug released. According to in vitro analysis, the free DOX was more effectively absorbed into tumor cells than the VLPs. In fact, PhMV‐based VLPs are greatly biocompatible, with elongated circulation times and a preference to accumulate in tumors. PhMV‐based VLPs could offer a greatly appropriate system in order to develop effective drug delivery system formulations. The anti‐tumor activity of VLPs was evaluated against a breast cancer (MDA‐MB‐231) xenograft tumor model in NCr nude mice. Results show that the tumor sizes in the control group grew rapidly, whereas in the PhMV‐PEG particle control group, tumor development was considerably inhibited. This could suggest that VLPs have potential to stimulate an innate immune response in the tumor microenvironment independently. In mice treated with DOX‐PhMV‐PEG particles, tumor growth was significantly inhibited and in some cases completely disappeared, indicating that the particles aggregated in the tumors released DOX on demand, which destroyed the tumor cells with high efficacy.^[^ ^98^ ^]^

### 5.4 HBc‐Virus‐Like Particle

Epilepsy is characterized by a transitory brain malfunction caused by a rapid aberrant discharge of excitatory neurons in the brain. 30% of epilepsy patients acquire antiepileptic drug resistance and experience uncontrollable seizures, referred to as refractory epilepsy.^[^ ^103^ ^]^ Epilepsy refractory to antiepileptic drugs is linked to the formation of the blood–brain barrier, which prevents them from entering the brain.^[^ ^101^ ^]^ Protein nanocages (NCs) have been discussed extensively as a viable drug delivery technique for clinical treatment because of their biocompatibility and biodegradability. In a series of protein NCs, the hepatitis B core protein (HBc) has been extensively studied for biomedical applications. HBc can be synthesized and self‐assembled using well‐known expression techniques, as well as modified using various peptides.^[^ ^104^ ^]^ As a delivery vehicle, an HBc VLP might provide several benefits, including preventing drug deterioration and improving drug delivery to specific sites. In addition, genetic engineering can transform HBc VLPs into hollow VLPs that lack undesired viral genes and hence are replication‐deficient and non‐infectious. Furthermore, brain tumor targeting efficacy of HBc VLP may also be improved by modification of various peptides.

**Figure 13:**
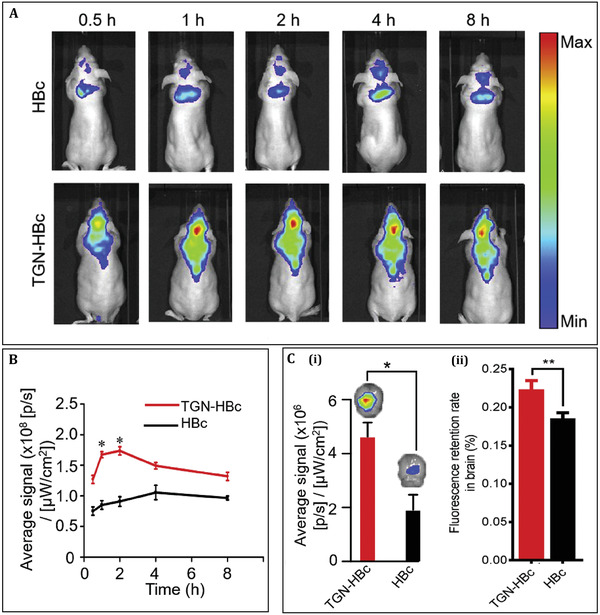
TGN‐HBc nanocages efficiently target the brain. A) In vivo imaging of Cy5.5‐labeled TGN‐HBc nanocages in the brain and HBc nanocages as a control at various time points. B) The fluorescence intensity of the brain region was used to quantify TGN‐HBc nanocages and HBc nanocages. C) The ex vivo brain images and fluorescence data from two mentioned groups (i) and the rate of fluorescence retention in brains of each group (ii). *p ˂ 0.05, **p ˂ 0.01). (*n* = 3). Reproduced with permission.^[^ ^105^ ^]^ Copyright 2020, Elsevier. Abbrevition: HBc: Hepatitis B core.

In order to facilitate the delivery of the epileptic drug, phenytoin, to the brain, Zhao et al. successfully developed a delivery system by inserting a brain targeting TGN peptide in to the nanocage of the HBc protein. The results from this study in pilocarpine‐induced epilepsy models demonstrate 2.4‐fold increase in selective brain tissue target and nearly 100 times improvement in the antiepileptic efficiency using this nanocage. Both in vivo mice and in vitro human neural cell studies established that TGN‐Hepatitis B core (TGN‐HBc) nanocages shows low cytotoxicity and high penetrating ability. Rather than disrupting the blood brain barrier, these effects are done by facilitating the brain target peptide TGN (**Figure** **13**). The result showed higher PHT accumulation in the brains of TGN‐HBc animals and the PTH concentration stretched about 92 ng g^−1^ in brain 1 h after the injection. These outcomes also revealed strong signal distribution in the liver and kidney, which showed that TGN‐HBc nanocages have major brain targeting efficiency.^[^ ^105^ ^]^

A dual‐targeted delivery system containing TGN brain targeting peptide and tumor vascular preferred ligand RGD was also developed using HBc protein–VLP delivery system for glioblastoma targeting. Yes‐associated protein (YAP) acts as a transcriptional co‐activator of the Hippo Pathway, which regulates cell proliferation and death. YAP was recognized in chicken as a binding protein of nonreceptor tyrosine kinase YES1. The interaction is arbitrated through a proline‐rich region of YAP and the SH3 domain of YES1.^[^ ^106^ ^]^ Glioma cell invasion and migration may be enhanced by overexpression of YAP. The incorporation of YAP siRNA into a dual‐targeted delivery system has the potential to cause apoptosis in tumor cells and prevent tumor invasion. Given the limited effects of siRNA, paclitaxel (PTX) co‐packaging could have some therapeutic potential. Although PTX is widely used as an anticancer agent, a limited amount of it reaches the brain. As a result, it has restricted anti‐tumor activity while having significant adverse effects at high doses. Based on these data, the particles of PTX/siRNA@TGN/RGD‐HBc VLPs were created by packaging the delivery system with PTX and YAP siRNA. The drug loading content of 23.28 ± 2.24% and an encapsulating efficiency of 76.09 ± 10.44% were reported for PTX@TGN/RGD‐VLP system. After 24 h at room temperature and pH 7.4, ≈40% of the PTX was released. The combination of chemotherapy and gene therapy showed potent synergistic anti‐tumor effects by increasing the apoptosis rate and inhibiting tumor invasion with limited cytotoxicity. Combination treatment using several approaches, including several targets and various therapeutic agents, could have beneficial therapeutic efficacy for invasive glioblastoma. These results showed that the use of carrier was nontoxic for this application and it could be used in a variety of drug delivery applications in the future.^[^ ^107^ ^]^

### 5.5 JgCSMV

Johnson grass chlorotic stripe mosaic virus (JgCSMV) empty shells were investigated as VLP nanoparticles for drug delivery application. In a recent study, JgCSMV‐VLPs were introduced as a cheaper and safer platform for the large‐scale nanoparticle synthesis. A recombinant VLPs of JgCSMV were produced by cloning of this virus coat protein (CP) gene into pBI121 vector and then introduced into Agrobacterium rhizogenes, which was explored as a nano‐container for DOX for administrating into human cells. In a tobacco hairy‐root expression system, Alemzadeh and colleagues reported that the in the absence of viral RNA, the JgCSMV‐CP coat can be expressed and in vivo test also confirms the assembling of recombinant protein into empty shells of VLPs.^[^ ^108^ ^]^ Researchers have examined the possibilities of employing the empty shells of icosahedral JgCSMV as carriers for drug delivery in this context. The particle form appeared to be compact at low pH, but swelling and opening of pores occurred as the pH increased due to structural transition. DOX loading inside the VLP core may benefit from porous structures and reversible mechanisms. The DOX can be encapsulated within the JgCSMV‐VLP by diffusing via the pores and electrostatically interact with glutamic acid and aspartic acid residues present inside the capsids. According to the results of this investigation, each VLP can encapsulate an average of 2500 DOX molecules.^[^ ^108^ ^]^

### 5.6 Q*β*

VLPs have attracted widespread attention in the nanomedicine field as carriers of various agents with different molecular functions with the possibility for precise cellular delivery, inspired by natural viruses' innate polyvalency and tissue tropism.^[^ ^109^ ^]^ The VLPs, which are derived from bacteriophage Qß (*Qubevirus durum*) have been extensively manipulated in order to be used in cell targeting, imaging, and immunology. In this regard, Crooke and colleagues developed a VLP‐macrolide conjugate platform for intracellular drug delivery in the situation of pulmonary infections. This study established the ability of macrolides to guide a VLP to the lungs, most likely by selective accumulation in pulmonary macrophages. Among all the ligands examined, azithromycin prompted the greatest macrophage uptake in vitro, and uptake was shown to be dependent on both the macrolide's orientation and density on the particle surface. In mice, azithromycin conjugation guided VLPs to the lungs, where they accumulated significantly within 2 h after systemic injection.

These findings indicate that this novel class of bio‐conjugates may represent an effective technique for pulmonary infection drug delivery.^[^ ^110^ ^]^

### 5.7 CPMV

The new treatment used by researchers designing Cowpea mosaic virus (CPMV) nanoparticles for human cancer therapy is eCPMV. eCPMV preserves all of CPMV's well‐known capsid proteinaceous structural properties, but without the RNA genomes. As a result, eCPMV is non‐replicative and thus non‐infectious in humans.^[^ ^111^ ^]^ CPMVs can hold molecules in their protein capsids for long periods, distinguish between cancerous and normal cells, be picked up and collect within tumor cells, and then release their cargo biomolecules until internalized.^[^ ^112^ ^]^ Because of these characteristics, CPMVs are very good at supplying toxic substances to tumor cells while missing the normal host cells, making them potent pharmacological vectors in cancer therapy.

**Figure 14:**
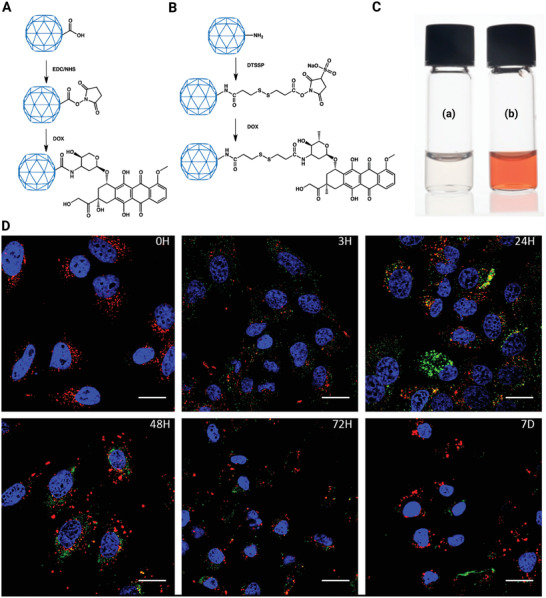
The conjugation process for preparing A) CPMV‐DOX and B) CPMV‐SS‐DOX. C) Aqueous suspensions of wild‐type CPMV (a) and CPMV‐DOX (b) are depicted schematically. D) Time course study using confocal scanning microscopy showing cellular uptake of CPMV (Green: CPMV, red: Endolysosomes, blue: nuclei). Reproduced with permission.^[^ ^113^ ^]^ Copyright 2013, American Chemical Society. Abbreviation: CPMV: Cowpea mosaic virus; DOX: Doxorubicin.

Efficient tumor entry, tumor cell internalization, and affecting of the loaded CPMVs to the endolysosome were achieved using CPMVs covalently loaded with the chemotherapeutic agent's DOX, but cargo release was delayed before the internalized CPMV particles were steadily metabolized by the tumor cells for just a few days.^[^ ^113^ ^]^ DOX was covalently conjugated using the EDC/NHS to the carboxylate groups of the native CPMV (**Figure** **14**A) to produce CPMV‐DOX, or amide bond was created using an eight‐atom spacer arm cross‐linker with a disulfide bond with amine group on the surface of CPMV to produce CPMV‐SS‐DOX (Figure 14B).

Tissue culture methods were used to assess the cell viability and drug effectiveness of CPMV‐SS‐DOX and CPMV‐DOX. CPMV nanoparticles in their natural state are biocompatible and do not seem to be cytotoxic (Figure 14). The cell‐killing ability of CPMV‐DOX conjugates was demonstrated. When analyzing CPMV‐SS‐DOX and CPMV‐DOX, they discovered major variations in cellular toxicity trends. CPMV‐SS‐DOX's effectiveness was similar to that of the free drug. Information shows that when DOX is bonded to CPMV through a disulfide bridge, the effective dose remains unchanged. This is due to a disulfide‐bridge dissociation in the culture medium enables doxorubicin from CPMV to be released before cellular uptake. Therefore, CPMV‐SS‐DOX resembles free DOX in its operation. However, targeting techniques may be used to enhance any design additionally. Cardiotoxicity is caused by the free drug, which limits its therapeutic use. Conjugation of a receptor‐targeted CPMV nanoparticle with a covalent amide bond is required to have tissue‐specific delivery benefits.

In general, as compared to free DOX, the CPMV‐DOX compound is a more cytotoxic cause against HeLa cells at low dosages, while the cytotoxic impact is delayed as the concentration is increased. Without the usage of cell‐specific targeting agents on the capsid, the CPMV nanoparticles are steered to the endolysosomal compartment of the cells. After all, the platform technology can be further developed to add targeting ligands that fit a patient's clinical profile, paving the way for the advancement of customized cancer medicines.^[^ ^113^ ^]^

### 5.8 TMV

Tobacco mosaic virus (TMV) nanoparticles with self‐replicating properties have frequently been applied in order to drug delivery; though, when recombinantly released, TMV proteins can combine into several states, such as simple monomers and micron‐long helical rods. To supplement the existing spherical‐ and rod‐shaped protein materials, it would be beneficial to create a new and secure morphology.

Disk‐shaped nanoparticles are an under‐appreciated but theoretically fascinating morphology for drug delivery. Disks may have an advantage over other morphologies in that they may fit into narrow spaces and have greater penetration into tumor cells.

They present a TMV dual Arg mutant (RR‐TMV) which assembles into dual disks that remain similar throughout environmental changes. Further, DOX conjugated with these disks at non‐native cysteine and N‐terminus of PEG for drug delivery, and cell absorption and toxicity were tested.

Protein‐based nanoparticles are often bonded to PEG polymers to minimize immunogenicity and improve serum constancy as they are designed in order to drug delivery. Researchers tried to use these robust RR‐TMV disks for drug distribution applications now that they had them. DOX, a commonly applied drug in order to nanoscale drug delivery, was compressed onto the hydrazide maleimide linker EMCH and conjugated to the RR‐TMV disks at the S123C location through maleimide chemistry.^[^ ^114^, ^115^ ^]^ The PDA‐coated TMV exhibited a shorter plasma circulation time and wider distribution to various organs, such as liver, spleen, and lungs than uncoated TMV particles. Though, the ELISA test showed that PDA‐coated particles connect two times lesser to anti‐TMV antibodies removed through particle injection than uncoated particles, proposing that the PDA coat allows elusion from systemic antibody surveillance. TMV‐PDA particles were also cleared from organs after 7 days. The slower tissue clearance of the coated particles causes them appropriate in order to theranostic applications.^[^ ^116^ ^]^

**Figure 15:**
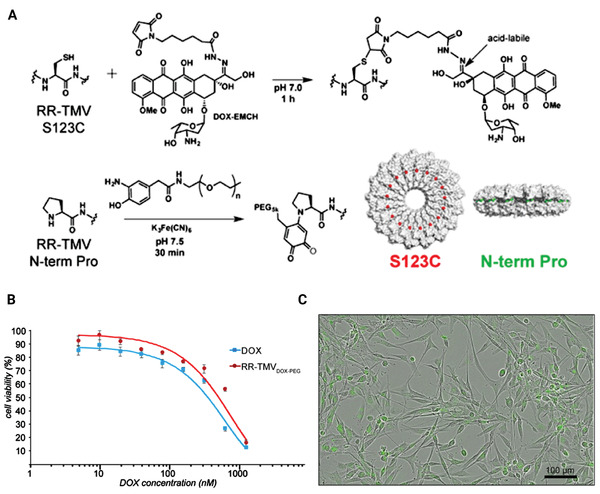
RR‐TMV _DOX_‐_PEG_ conjugates are characterized. A) RR‐TMV disks were loaded with EMCH DOX at their S123C positions and aminophenolPEG5k at their N‐terminal prolines. B) Significant cell death observed after 72 h of incubation of U87MG glioblastoma cells with varying concentrations of RR‐TMVDOX‑PEG conjugates. C) U87MG cell uptake of RR‐TMVDOX‑PEG conjugates at 48 h. Reproduced with permission.^[^ ^117^ ^]^ Copyright 2016, American Chemical Society. Abbreviation: DOX: Doxorubicin; TMV: Tobacco mosaic virus; PEG: Polyethylene glycol.

The RR‐TMV_DOX‐_ _PEG_ conjugates' drug delivery ability was then tested in vitro. When DOX‐treated and RR‐TMV_DOX_‐_PEG_‐treated cells were compared to cell controls after 3 weeks of incubation, substantial cell death was detected (**Figure** **15**). The relaxed‐release profile of the hydrazone bond between EMCH and DOX is most likely responsible for the disparity in cell viability bends among RR‐TMV_DOX_‐_PEG_ and DOX alone. As a result, RR‐TMV disks offer an exciting new nanostructure that will add to the form catalog of protein‐based biomaterials in order to in vitro drug delivery. Upcoming research will concentrate on understanding the RR‐TMV disks' special assembly state in addition to their biodistribution and transmission efficacies.^[^ ^117^ ^]^

### 5.9 CCMV

**Figure 16:**
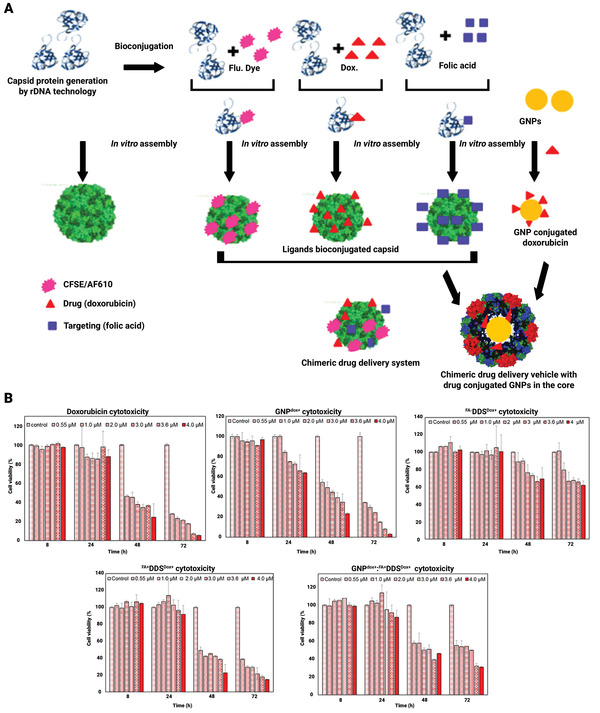
A) The development of the chimeric drug delivery mechanism is depicted schematically. The CCMV virus's customized capsid proteins were folic acid, dye, and DOX separately. The right combination of these ligand bioconjugated capsids was constructed with DOX conjugated AuNPs at its center to create nanoparticulate targeted delivery vehicles. B) Cytotoxicity of various drug delivery system formulations toward MCF7 cells by XTT Assay. Reproduced with permission.^[^ ^118^ ^]^ Copyright 2016, Nature. Abbreviation: DOX: Doxorubicin; CCMV: Cowpea chlorotic stripe mosaic virus.

For effective drug delivery in cancer cells, ligands bioconjugation was used to create targeted nano‐delivery carriers from genetically engineered cowpea chlorotic mottle virus (CCMV) capsid. To achieve effective in vitro assembly lacking the usual payload, the capsid's RNA‐binding and hexamer‐forming domains were selectively removed by genetic engineering. To improve the stabilization, drug packing, and continuous release of a drug, the cavity of the nano‐scaffold was filled with DOX covalently attached gold NPs. At a pH of 4–8, the chimeric structure remained constant. This genetically engineered nano‐scaffold method displayed greatly detailed receptor‐mediated internalization (targeting) and high cytotoxicity toward folate receptor‐positive MCF7 cell lines. The current device could provide a customizable nano‐scaffold‐based framework for the development of cancer chemotherapeutics. In vitro assembly of genetically engineered CCMV capsid on DOX conjugated AuNPs was used to create a chimeric multipurpose drug delivery system, as seen in **Figure** **16**. The modified capsid proteins were made in *Escherichia coli* (*E. coli*) and bioconjugated with folic acid and DOX in the purposeful form separately. In vitro processing was applied to encapsidate DOX‐doped AuNPs in the inner cavity of these functionalized capsid proteins to improve the delivery vehicle. On MCF7 cells, the cytotoxicity of DOX conjugated reformed delivery carriers (GNPdox+, FADDSdox+, FA+DDSdox+, and GNPdox+:FA+DDS dox+) was compared to that of equimolar pure doxorubicin. Based on the free DOX concentration, 50–60% cellular cytotoxicity was detected after 48 h and 80–90% after 72 h. Pure DOX, on the other hand, demonstrated very low cytotoxicity up and about to 4.0 µm. The synthesis of targeted bioengineered DOX delivery vehicles to FR expressed cancer cells is demonstrated in this research (MCF7). Assembly of genetically modified and ligands conjugated, capsid proteins of CCMV developed the vehicle. To improve drug loading capability and boost drug efficiency, DOX conjugated AuNPs are placed in the internal cavity of these vehicles. During the in vitro release trial, this vehicle demonstrated selective DOX release in cellular lysosome mimicking situations. In FR‐positive cells, these carriers demonstrated uptake and cytotoxicity (MCF7).^[^ ^118^ ^]^

### 5.10 CCMV with Heparin

Heparin is a glycosaminoglycan polysaccharide commonly applied as an anticoagulant in surgical procedures cases.^[^ ^119^ ^]^ The CCMV capsid is a highly explored VLP as it can be grown in large quantities and undergoes swelling in various ionic strengths making it a popular choice for cargo packing. They discovered that heparin‐required peptide modified CCMV capsid proteins at physiological pH levels show unique heparin binding. Based on DLS measurements and TEM representation, co‐assembly with heparin capsids showed the size of about 20 nm. These findings suggest that VLPs are promising heparin‐binding materials, particularly when the connecting is adapted to physiological conditions and after extensive hematologic studies. Capsid creation relies on the amount of encapsulated compound, and great cargo ratios limit capsid development. The HBP in issue was chosen because of its great discernment and affinity for heparin, reducing off‐target connecting in complex biological settings.^[^ ^120^ ^]^ The SrtA‐based approach was used to conjugate HBP to the CP N‐terminus. Finally, they demonstrated that CCMV capsid proteins functionalized with a heparin‐binding peptide at physiological pHs display unique heparin binding. Due to DLS and TEM classification, co‐assembly with heparin formed *T* = 1 capsids. The capsid formation was further confirmed by fast protein liquid chromatography findings, which showed a heparin concentration influence on capsid development. These findings suggest that VLPs are hopeful heparin‐binding materials under physiological relevant conditions.^[^ ^121^ ^]^

### 5.11 PhMV

Physalis mottle virus (PhMV)‐VLPs were genetically modified to show diagnostic and immunogenic epitopes in previous studies.^[^ ^122^ ^]^ The surface of PhMV was investigated, and protocols for detailed functionalization of these surfaces with various agents were established. PhMV was modified with dyes, medications, and photosensitizers using a library of functionalization procedures, such as conjugation and noncovalent binding. Flow cytometry and confocal microscopy were used to classify the functionalized PhMV nanoparticles, and their cytotoxic efficiency was checked in a variety of cells.^[^ ^122^ ^]^

The VLPs were packed with fluorescein, rhodamine B, MTX dihydrochloride, or DOX hydrochloride. The VLPs were incubated overnight at room temperature with a molar extra of cargo molecules per atom. Since the virus is primarily stabilized through protein–protein interactions, drug encapsulation by particle disassembly and assembly is not feasible in the case of PhMV. As a result, they devised an infusion procedure for loading cargo into the VLPs.

In this study, they exposed MDA‐MB‐231 breast cancer cells and ovarian cancer to a variety of concentrations of free DOX and equal concentrations of DOX in the DOX‐PhMV particles to see whether DOX maintained its cytotoxic function in the particles. Since DOX chemotherapy is applied to the first‐line and adjuvant care in women with breast or ovarian cancer, they preferred breast cancer and ovarian. Increased bacterial fermentation capability could be used to scale up the output of PhMV‐based VLPs, but expression in plants could achieve much greater scalability. PhMV does not replicate in mammals but is compatible and degradable as opposed to mammalian virus‐based structures, providing an extra layer of protection. The surface of PhMV particles will be decorated with shielding molecules to avoid clearance or targeting ligands to guide the particles to particular tissues and cells in future studies.^[^ ^122^ ^]^

### 5.12 MrNV

Hyperthermia therapy is a method of cancer treatment that involves exposing cancer patients' tumor tissues or other body parts to various temperatures.^[^ ^123^ ^]^ *Macrobrachium rosenbergii* nodavirus (MrNV) is an icosahedral virus with several copies of the viral capsid protein, where each capsid contains polypeptide of 371 amino‐acids. In *E. coli*, the chimeric capsid protein encapsidates RNA molecules by self‐assembling into a VLP. MrNVLP is the name of the VLP.^[^ ^124^ ^]^

**Figure 17:**
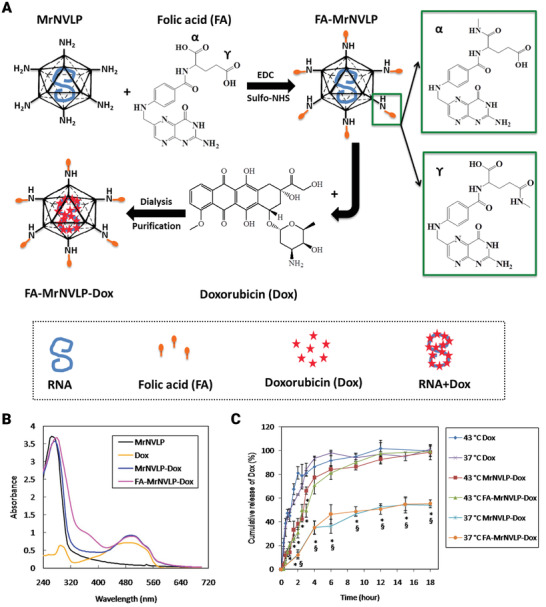
A) Focusing on the VLPs of *Macrobrachium rosenbergii* (*M. rosenbergii*) nodavirus, a schematic image of a drug delivery mechanism is seen (MrNVLP). Applying EDC and *N*‐hydroxysulfo‐succinimide, carboxylic acid groups of folic acid were conjugated with the amines of lysine residues found on the surface of MrNVLP (sulfo‐NHS). Via associations with the RNA molecules encapsidated within the nanoparticles, DOX biomolecules were injected into the cavity of FA‐conjugated MrNVLP. B) UV–vis spectra of the virus‐like particle of *M. rosenbergii* nodavirus (MrNVLP), free doxorubicin (DOX), DOX‐loaded MrNVLP (MrNv‐DOX), and DOX‐loaded‐and‐folic acid (FA)‐conjugated MrNVLP (FA‐MrNVLP‐DOX). C) Drug release profiles of DOX, MrNVLP‐DOX, and FA‐MrNVLP‐DOX for the first 18 h at 37 and 43 °C. Reproduced with permission.^[^ ^125^ ^]^ Copyright 2019, Nature. Abbreviation: FA: Folic acid; EDC: 1‐ethyl‐3‐(3‐dimethyl aminopropyl) carbodiimide hydrochloride; Sulfo‐NHS: N‐hydroxysulfosuccinimide; DOX: Doxorubicin.

DOX is extremely toxic to healthy cells, particularly those in the cardiovascular system. As a result, a new drug delivery mechanism that can target DOX to targeted cells while having fewer side effects on normal cells is highly demanded. A DOX delivery system was developed based on a new thermally sensitive carrier based on MrNVLP. In this research, Folic acid ‐MrNVLP‐DOX (FA‐MrNVLP‐DOX) (**Figure** **17**A), an FA‐conjugated MrNVLP filled with DOX, which is rich in HT‐29 cells and DOX is released at 43 °C. This study's multifunctional nanomaterial displayed precisely targeted distribution and thermally regulated release of DOX. FA‐MrNVLP‐DOX and MrNVLP‐DOX had entrapment efficiency of 5.06 ± 0.29% and 5.47 ± 0.13%, respectively (Figure 17B). MrNVLP‐DOX and FA‐MrNVLP‐DOX had loading performance of 2.1 ± 0.1% and 2.3 ± 0.2%, respectively. After 12 h at 37 °C, nearly half of the DOX was released from the nanoparticles, and the device only reached a full overall release of about 80% after 3 days. At 43 °C, however, roughly half of the DOX was released from the nanoparticles, and nearly all of the Dox was released within 1 day (Figure 17C). They developed a significant method in order to directly load DOX into MrNVLP in this study. FA conjugation to the VLP improved cellular uptake and aggregation of DOX in FR‐rich HT29. Furthermore, FA‐MrNVLP‐DOX improved DOX toxicity and apoptotic activity in HT29 cells. Other tumor‐targeting ligands could be expressed on MrNVLP using suitable cross‐linkers in addition to FA. MrNVLP's long‐term release profile and virtually complete release of all the loaded DOX at hyperthermia temperatures show that it may be a good choice for a thermally sensitive drug delivery system (Figure 17).^[^ ^125^ ^]^ **Table** **2** lists some genetically engineered viruses that are employed in the delivery of drugs for treatment of different diseases.

**Table 2:** Some examples of genetically engineered viral targeted for drug delivery applications

Viral	Shape	Family	Drug	Disease	Ref.
P22‐SP	Icosahedral	Podoviridae	Ziconotide	HIV and cancer	[129]
P22 WB	Icosahedral	Podoviridae	Bortezomib (BTZ)	Cancer	[9]
tHBcAg	Icosahedral	Hepadnaviridae	Doxorubicin	Colorectal and cervical cancer	[130]
Reovirus	Icosahedral	Reiviridae	Doxorubicin, epirubicin, topotecan	Triple‐negative breast cancer	[131]
MCF7 specific fusion phage	Rode shape	Bacteriophages	Doxorubicin	Breast cancer	[132]

### 5.13 P22‐SP

Ziconotide (Prialt), the first non‐opioid analgesic identified in the venom of the sea snail *Conus magus*, is being used to treat medical conditions in HIV and cancer patients. The peptide was encased in a viral container and administered across the BBB using a trojan horse approach, which has previously been described as a viable replacement for intrathecal injection in order to deliver ziconotide.^[^ ^126^ ^]^

A strategy for inducing disintegration of a nano container made from the viral capsid of the *Salmonella typhimurium* bacteriophage P22 is disclosed in physiologic conditions. The P22 capsid is an icosahedral lattice that self‐assembles when enough copies of the P22 scaffold protein are present.^[^ ^127^ ^]^

The Douglas group developed a strategy to package arbitrary gene yields into the P22 capsid shell by using standard recombination procedures to build a fusion protein including an uninformed carrier protein attached to the P22‐SP C‐terminus via a thrombin cleavage site.^[^ ^128^ ^]^ The fluorescent proteins EGFP and mCherry, the enzyme alcohol dehydrogenase D, and the HIV and cancer drug ziconotide were all loaded into the P22 capsid in this way.^[^ ^129^ ^]^

### 5.14 P22 WB

P22 viral capsids have recently been employed as platforms in order to generate an effective delivery vehicle using genetic and chemical engineering techniques. Catechol ligands are linked to the inner surface of the P22 viral capsid for boronic acid‐diol complexation encapsulation of the anticancer medication bortezomib (BTZ).^[^ ^9^ ^]^ Fluorescent cell imaging is used to demonstrate effective delivery of produced P22 viral capsid composites, and a cell survival assay is used to assess the efficiency of delivered P22 viral capsid nanoparticles. 420 identical capsid subunits were initially organized into an icosahedral procapsid structure with the aid of around 300 copies of internal scaffolding proteins. The P22 procapsid was heat‐treated at 75 °C for 15 min, resulting in the formation of a waffle‐ball (WB) capsid with twelve 10‐nm holes along the fivefold axes, resulting in a hollow nanocomposite. P22 WB capsids have genome‐free hollow structures with ample space inside their cavity for small chemotherapeutic medicines and/or diagnostic probes. BTZ was selected as an anticancer medication cargo to release to specific cancerous cells because it is a dipeptide boronic acid analog that forms a continuous complex with catechol ligand at neutral or alkaline pH through the boronic acid‐diol complexation.^[^ ^9^ ^]^

### 5.15 tHBcAg

Hepatitis B VLNP is one of the most well‐studied VLNPs. It is made up of 180 or 240 viral core antigen subunits (HBcAg). The arginine‐rich region at the C‐terminus of a truncated HBcAg mutant (tHBcAg) similarly self‐assembles into icosahedral VLNPs. DOX was covalently bonded to the exterior surface of these NPs through carboxylate groups by studying the chemistry of truncated hepatitis B core antigen (tHBcAg) VLNPs. Each tHBcAg VLNP has about 1600 DOX molecules attached to it. The results showed that as compared to free DOX, the dual conjugated tHBcAg VLNPs boosted DOX accumulation and absorption in human colorectal and cervical cancer cell lines, resulting in greater DOX cytotoxicity toward these cells.^[^ ^130^ ^]^

### 5.16 Reovirus

Triple‐negative breast cancer is a kind of breast cancer that has a higher recurrence rate, a lower life expectancy, and fewer therapy options for patients. Mammalian orthoreovirus (reovirus) selectively infects and kills changed cells, and a serotype 3 reovirus is being evaluated in clinical trials as an oncolytic therapy against a number of cancers.

The nonfusogenic mammalian orthoreovirus (reovirus) is a dsRNA virus that is not enclosed. The effectiveness of a serotype 3 reovirus (Reolysin) against a range of malignancies is being tested in phase I and II clinical studies. These findings reveal that recombinant viruses with a unique genetic makeup created via forwarding genetics and topoisomerase inhibitors infect and destroy triple‐negative breast cancer cells more effectively. In this study, the researchers developed two novel reoviruses that more successfully infect and kill triple‐negative breast cancer cells. They observed that reovirus infection increases in the presence of DNA‐damaging substances and kills breast cancer cells.^[^ ^131^ ^]^

## 6 Immunogenicity, Protein Corona, Clearance, and Biodistribution

Engineered nanomaterials embrace potential to an extensive range of uses in medicine. Though, Interaction of nanomaterials with biological agents improve their safety. One of the most important points is the immunogenicity of nanomaterials in medical application. When nanoparticles enter the human body, they can known through immune system (innate and adaptive). They can stimulate a cascade leading to activation of cell, as well as release of different chemokines and cytokines. In fact, interactions of nanoparticles with the immune system cause an allergy and immunogenicity, including innate and adaptive immune responses. Inherent NP characteristics may affect their immunotoxicity.^[^ ^133^, ^134^ ^]^ As a result, it is important to evaluate the safety response against nanomaterials. Nanoparticles have high surface area, enhancing the probability of surface associated reactions at bio interfaces. For example, an enhancement in the number of electron donor or acceptor active sites tips to the creation of oxygen superoxide radicals which produce ROS. These free radicals can overcome the antioxidant defenses and lead toxicity.^[^ ^135^ ^]^

However, engineering organic materials, such as ELP and SELP, show low‐immunogenic biopolymer with wide confirmation supporting its application as a carrier for efficient drug delivery systems than other organic materials. Cappello et al. showed low immunogenicity of a SELP in rabbits. Here, the SELP polymer was injected for 6 and 8 weeks (0.5 mg). No reactivity of the samples was revealed in order to binding to (VPGVG)_8_ sequence.^[^ ^136^ ^]^

Viral vectors, such as VLP, seem to be the most favorable model in biomedical studies. However, instability and immunogenicity are among their limitations.^[^ ^137^ ^]^ Attempts are being made to produce engineered VLPs with lower immunogenicity and higher efficiency. For example, researchers use *E. coli*‐based cell‐free protein synthesis to quickly create HBc proteins that self‐assemble into VLPs. To increase nanoparticles stability, covalent disulfide bonds were presented in the VLP. Negative charges on the HBc VLP surface were also decreased to increase conjugation. This stabilized HBc VLP showed no immunogenicity in mice, representing high promise in order to clinical uses and drug delivery systems.^[^ ^137^ ^]^

However, drug delivery using these genetically engineered carriers has yet to be transformed destructively to apply in clinical applications. In fact, novel concepts and perspectives are required to development genetically engineered nanocarriers into the medicine; though, their various pre‐clinical uses propose that they may offer a great novel assay in order to producing nanomedicines.

As mentioned, nanomaterials can be detected by immune cells, although one of the important points is to prevent them from being cleared from the host, which is especially important in nanomedicine. In fact, if the goal is to enter tumor cells in the brain, unplanned clearance by the immune system should be avoided. Also, if the target is the target immune system, especially for vaccination, nanoparticles should be designed properly. One way is PEG nanoparticles surface location to prevent nonspecific clearance by the reticuloendothelial system.^[^ ^138^ ^]^ Engineering polymers, such as SELP polymers proposed high potential as drug delivery system. Quick clearance can create a strong barrier that decreases the efficiency of pharmacological agents in the bladder. In fact, in order to decrease Semi‐synthetic glycosaminoglycan ethers clearance and increase its accumulation in the bladder, temperature‐responsive SELP polymer‐based system can be used.^[^ ^139^ ^]^

One of the ideas about ELPs is that with an enhancement in MW, ELPs have an improved half‐life; smaller ELPs will be quickly cleared through the kidneys, although larger ELPs will remain in blood, providing more time to accumulate. For example, a study was performed in SKH1 Elite mice to detect the effects of MW on ELP. Here, they used five ELP proteins with different MW (25–86 kDa). The results showed that an increase in MW caused slower plasma clearance. The results of the half‐life study showed that the half‐life of the smallest protein, such as ELP‐63 (25 kDa), was short. However, the biggest protein, ELP‐223 (86 kDa), showed a half‐life of about 16 h, with a 20 times enhancement.^[^ ^140^ ^]^

In order to perform an effective cancer therapy, it is necessary to prevent the rapid clearance of the VLN during blood circulation. VLN is also needed for effective accumulation in tumor tissue after systemic administration. Mice treated with VLN showed a greater residual concentration at 48 h after intravenous injection. In fact, VLNs have a longer half‐life (14 h) where the lipid layer containing PEG encapsulation can effectively prolong VLN circulation. Also, due to the increase in fluorescence intensity in tumors of VLN‐treated mice compared to other groups, this increased accumulation of nanocarriers is mostly ascribed to PEG‐DSPE components at the VLN level and permeability effects.^[^ ^102^ ^]^

Biodistribution studies have displayed that the uptake of spherical nanoparticles is favored over nonspherical nanoparticles. The ratio of internalization of nonspherical materials depends on their angular trend with admiration to the cell membrane. Though spherical structures are applied usually for tumor targeting according to their facile synthesis assays, highly efficient tumor targeting has been observed in nonspherical nanostructures such as CNTs.^[^ ^141^ ^]^

Studies also show how MW impacts biodistribution and renal localization of the therapeutic carriers such as ELP and ELPs. Biodistribution studies exhibited that ELPs in appropriated size accumulated in kidneys.^[^ ^72^, ^140^ ^]^ Enhancement in size of ELP caused slower clearance in rodent pregnancy model.^[^ ^142^ ^]^ In previous studies, the capability of ELP to enter the CNS through intranasal administration and biodistribution of them has been revealed. The fusion of some agents such as Tat or SynB1 can be changed the biodistribution of ELP, reducing the CNS accumulation after intranasal administration. The addition of CPPs to ELP enhanced their maintenance in the nasal epithelium. These outcomes propose that ELP may show an effective CNS delivery carrier without modification and that the addition of a CPP considerably affects biodistribution.^[^ ^143^ ^]^ Various type of engineering viruses escape the immunogenicity response and offer benefits in nanomedicine and drug delivery according to monodispersed size distribution. Among them, CCMV capsids were subjugated to advance of drug delivery vehicles according to great stability at acidic pH environment.^[^ ^144^ ^]^ In addition, some studies with administered CCMV showed wide biodistribution with no toxicity and quick clearance in a nonpathogenic mode.^[^ ^145^ ^]^

**Figure 18:**
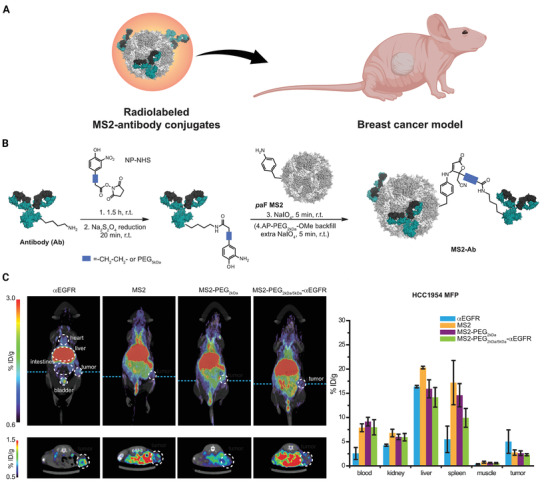
A) Biodistribution study of antibody‐MS2 capsid conjugates in cancer models. B) Formation of MS2‐antibody conjugates. C) In vivo biodistribution of Cu‐labeled MS2 in nude mice with HCC1954 orthotopic breast cancer tumors. PET scans were attained at 24 h after injection. Biodistribution studies were done 24 h after injection through gathering the main organs. The error bars display the standard deviation from samples. Reproduced with permission.^[^ ^146^ ^]^ Copyright 2016 American Chemical Society.

MS2 VLPs can carry up to 170 copies of drugs; the great amount of pharmacological agents accumulated in the tumor could offer a major therapeutic effect. MS2 VLPs can show proteins on their surfaces with the cell targeting. This phenomenon is possible by binding anti‐epidermal growth factor receptor (EGFR) antibodies to MS2 VLPs to target EGFR on breast cancer cells in the HCC1954 cell lines. Besides, MS2 is a vigorous platform that showed a slow clearance profile. The MS2 VLPs showed tumor‐to‐muscle ratios in the range of 2.5–4.5, showing precise uptake in the tumor than muscle tissue. The high accumulation of particles has showed tissues in the spleen and liver (**Figure** **18**).^[^ ^146^ ^]^

Nanoparticles can interact with various molecules after arriving into body. This outcomes in the creation of the protein corona.^[^ ^147^, ^148^ ^]^ Protein corona has a key role in making the nanoparticles simply known through the innate immune system, causing their rapid clearance through phagocytic cells. The protein corona changed the cytotoxicity and drug delivery efficiency of nanomaterials.^[^ ^149^ ^]^ However, they can cause the destabilization and accumulation of particles. These challenges cause the protein corona to prevent effective targeting.^[^ ^150^ ^]^ Besides, due to the toxicity of metal nanomaterials get up from the reactivity of their surfaces, the coating of nanomaterials through a protein corona effects a decrease in their cytotoxicity of them, avoiding direct penetration of metal nanomaterials crossways the cell membrane. For instance, the existence of a protein corona decreases the aggregation of spherical gold‐based nanomaterials.^[^ ^151^ ^]^

## 7 Challenges, Principal Limitations, and Future Direction

Aside from the numerous advantages of genetic modification, there are also some technical barriers in transferring these therapies to clinical therapy, particularly in terms of precision, effectiveness, tedious extraction process, controlling the morphologies of nanoparticles, and monodispersity in solution phase and delivery. To meet these obstacles, scientists will require in‐depth knowledge of the molecular basis of malignancies, particularly heterogeneous solid tumors, as well as properly developed genome editing platforms in pre‐clinical trials.

There are several biomolecules, capping and reducing agents that may be involved in the production and stability of nanoparticles by organisms; additional research is required to determine the mechanistic elements of such biological NP synthesis. The large‐scale, economic, and environmentally acceptable bio‐production of NPs with appropriate sizes, morphologies, and dispersity continues to be a challenge.^[^ ^152^ ^]^ A future task in this research will be investigating various reaction circumstances and genetic alterations to extend the sorts of metal nanoparticles that can be bio‐produced by improving reaction kinetics and developing novel techniques under neutral, non‐hazardous, room temperature settings. In the case of bacteria, for example, studies to increase biological heavy metal absorption have focused on enhancing uptake from the periplasm into the cytoplasm of Gram‐negative bacteria.

Biosystems have demonstrated natural control over the deposition and structure of inorganic materials and bio‐compounds, giving rise to biomimetic methods to the production of inorganic nanomaterials. Despite the availability of genetic tools/approaches and well‐organized manipulation methodologies, genetic engineering in organisms to create nanomaterials and nanoparticles has not been thoroughly researched. Because of the cell wall, which acts as a barrier to external biomolecule delivery, suitable and well‐organized genetic transformation in plants remains a significant issue.

One of the most challenging issues for the forthcoming use of genetic manipulation, will be the establishment of effective and secure techniques to deliver genetic editing elements not only to cancer cells ex vivo but to somatic cells in vivo. Considering their ease and safety, the relatively low delivery effectiveness of such non‐viral administration techniques restricts their therapeutic potential in vivo.^[^ ^153^ ^]^ In contrast, viral vectors with great delivery efficiency (such as retroviruses, lentivirus, and adenovirus) have been authorized for clinical usage.^[^ ^154^ ^]^ Despite the possibility of accidental alterations and the availability of safety margins, viral delivery techniques have proved to be the most successful system for delivering plasmid‐based nucleic acids to mammalian cells in vitro and in vivo.^[^ ^155^, ^156^, ^157^ ^]^

In recent years, significant scientific progresses have been made in the application of genetically engineered organisms for drug delivery via nanocarriers due to their ecofriendly, greener, high‐throughput, versatile nature, capability to modify their structural changes, and synthesis of nanomaterials and nanoparticles, even in an industrial scale.

This review has highlighted numerous genetically engineered organic, inorganic, and viral‐based nanocarrier designs augmented for targeted and therapeutic delivery to overcome the biological barriers across patient inhabitants and ailment. It seems that for the effective production of the nanoparticulate system via genetically engineered organisms, they should be tolerant to metal harmfulness, multiply promptly in economic media, produce high yields, and be engineered using the genetic engineering approach. Biomedical applications of genetically engineered nanomaterials are on the horizon, including drug delivery applications, synthesis of genetically engineered hydrogels for drug delivery applications, sutures, coating of wounds, artificial tendons, adhesive, and sealants for spinal disc repairs.^[^ ^158^ ^]^ Since the 1980s, genetically engineered organisms have appeared as one of the pillars of biomedical research. For example, genetically altered animal models of human genetic disease aided scientists in examining novel therapies and investigating the potential role of aspirant risk aspects and transformers of diseased factors. Genetic alterations of humans using gene therapy are also becoming a treatment option for numerous diseases ranging from disorders related to metabolisms to cancer.^[^ ^102^ ^]^

A wide variety of nanostructures have been modified using genetically engineered techniques. Furthermore, genetic engineering of bio‐based nanoparticles has significantly extended the appropriate areas of drug delivery systems. Later engineering of nanoparticulate systems can change the abilities not just by modifying structures, size, and shape of nanoparticles but also by removing the underprivileged characteristics and adding required skills.^[^ ^22^ ^]^ Biomaterials with specific flexibility make this remarkable engineering feasible. Therefore, nanomaterials that are hereditarily engineered with several capabilities are creating a substantial impact on the treatment of various diseased conditions and imaging technology.

The importance of genetic engineering of nanoparticles for forthcoming research is in the advancement of the techniques to resolve anti‐genicity and escape from the mononuclear phagocytic system since the clearance from the mononuclear phagocytic system leads to the accumulation of larger nanoparticles in the liver and pancreases.^[^ ^22^ ^]^ The success in incorporating nanoparticles in vivo is considerably less than those experienced in vitro. Genetic application of this approach to the nanoparticulate system would facilitate investigators to modernize the advancement of innovative targeted drug delivery nanoformulations with the help of target–ligand interactions that occur naturally. This review paper could open a new surge of biomimetic nanomedicine with finely crafted functionalities.

## Conflict of Interest

The authors declare no conflict of interest.
